# The synthesis of higher-carbon sugar alcohols via indium-mediated acyloxyallylation as potential phase change materials

**DOI:** 10.1007/s00706-023-03136-6

**Published:** 2023-12-16

**Authors:** Markus Draskovits, Nina Biedermann, Marko D. Mihovilovic, Michael Schnürch, Christian Stanetty

**Affiliations:** Institute of Applied Synthetic Chemistry, TU Wien, Vienna, Austria

**Keywords:** Carbohydrates, Indium, Ozonolysis, Aldehydes, Acyloxyallylation, Phase change materials

## Abstract

In recent years, sugar alcohols have gained significant attention as organic phase change materials (PCMs) for thermal energy storage due to their comparably high thermal storage densities up to 350 J/g. In a computational study, outstandingly high values of up to ~ 450–500 J/g have been postulated for specific higher-carbon sugar alcohols. These optimized structures feature an even number of carbon atoms in the backbone and a stereochemical configuration in which all hydroxyl groups are in an 1,3-*anti*-relationship, as found in the natural hexitol d-mannitol. However, these *manno*-configured higher-carbon sugar alcohols have not been experimentally investigated as PCMs yet and described synthetic routes are elaborate multiple steps syntheses. Therefore, we aimed to synthesize sugar alcohols of the *manno*-series with a concise synthetic protocol based on the indium-mediated acyloxyallylation (IMA) of aldoses. En route the C2-epimers were easily accessible, namely *gluco*-configured sugar alcohols, bearing one set of hydroxyl groups in a suboptimal 1,3-*syn*-realtionship. The synthesized compounds were found to possess thermal properties consistent with the predicted values, and the “perfect” higher-carbon sugar alcohol with eight carbon atoms was found to have indeed an outstanding high latent heat of fusion of ~ 380 J/g with a melting point of 260 °C.

**Graphical abstract:**

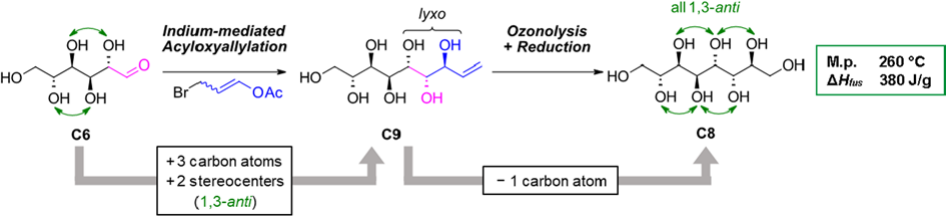

**Supplementary Information:**

The online version contains supplementary material available at 10.1007/s00706-023-03136-6.

## Introduction

In the last decades, the storage of energy especially in the form of thermal energy has become of significant importance with latent heat storage systems, namely phase change materials (PCMs), playing a major role [1]. Within PCMs, both organic and inorganic materials have gathered attention for their potential in thermal energy storage applications. Inorganic PCMs, such as salts, salt hydrates, and metals, have been favored in the past due to their high thermal conductivity and large heat storage capacities. However, inorganic PCMs often suffer from issues related to corrosion, phase segregation, and volumetric expansion during phase transitions, which can limit their practicality in some applications. In contrast, organic PCMs, including paraffin waxes and fatty acids with capacities in the range of 150–250 kJ/kg, offer advantages such as low toxicity and tunable phase change temperatures [1]. In recent years, sugar alcohols have gained more attention within organic materials when it comes to their application as PCMs [2–6] as a new field in addition to their wide usage in the food [7] and pharmaceutical industry [8]. Especially the sugar alcohols *meso*-erythritol (C4) and d-mannitol (**1**) (C6), among other natural sugar alcohols, have been intensively investigated as PCM candidate substances due to their relatively high thermal storage densities of above 300 J/g. For practical use, research has further focused on the encapsulation of sugar alcohols [9–11], among other techniques for organic-based PCMs [12], to improve their thermal stability, overcome disadvantageous melting and crystallization behavior like, supercooling phenomena, and also low thermal conductivity. In Table 1, the physical properties (melting temperature and latent heat of fusion) of selected examples for organic and inorganic key PCMs with industrial applications are presented.

**Table 1:** Selected examples for key PCMs used for application within different temperature ranges [13]

Temperature range	Material	Type	Melting temperature/°C	Heat of fusion/J/g	Application
Low (− 20 to 5 °C)	ClimSel™ C-18 (NaNO_3_/H_2_O)	Inorganic (salt hydrate)	− 18	306	Freezer
	Paraffin RT5 (mixture of C12-C18 paraffins)	Organic	5	198	Food packaging
Middle–low (5–40 °C)	Capric acid/lauric acid (65:35)	Organic	19.1	35.2	Wallboard
	48% CaCl_2_ + 4.3% NaCl + 0.4% KCl + 47.3% H_2_O	Inorganic (salt hydrate)	26–28	188	Floor heating
Middle (40–80 °C)	Lauric acid	Organic	42.5	211.6	Solar assisted thermal storage
	Paraffin wax 56	Organic	56	226	Solar stills
	Palmitic acid	Organic	61.3	186.1	Solar assisted thermal storage
High (80–200 °C)	Mg(NO_3_)∙6H_2_O	Inorganic	89	162	Solar cooker
	Erythritol	Organic	117.7	339.8	Solar cooling
	Adipic acid	Organic	152	275	Thermal storage
	d-Mannitol	Organic	165	300	Thermal storage

**Fig. 1:**
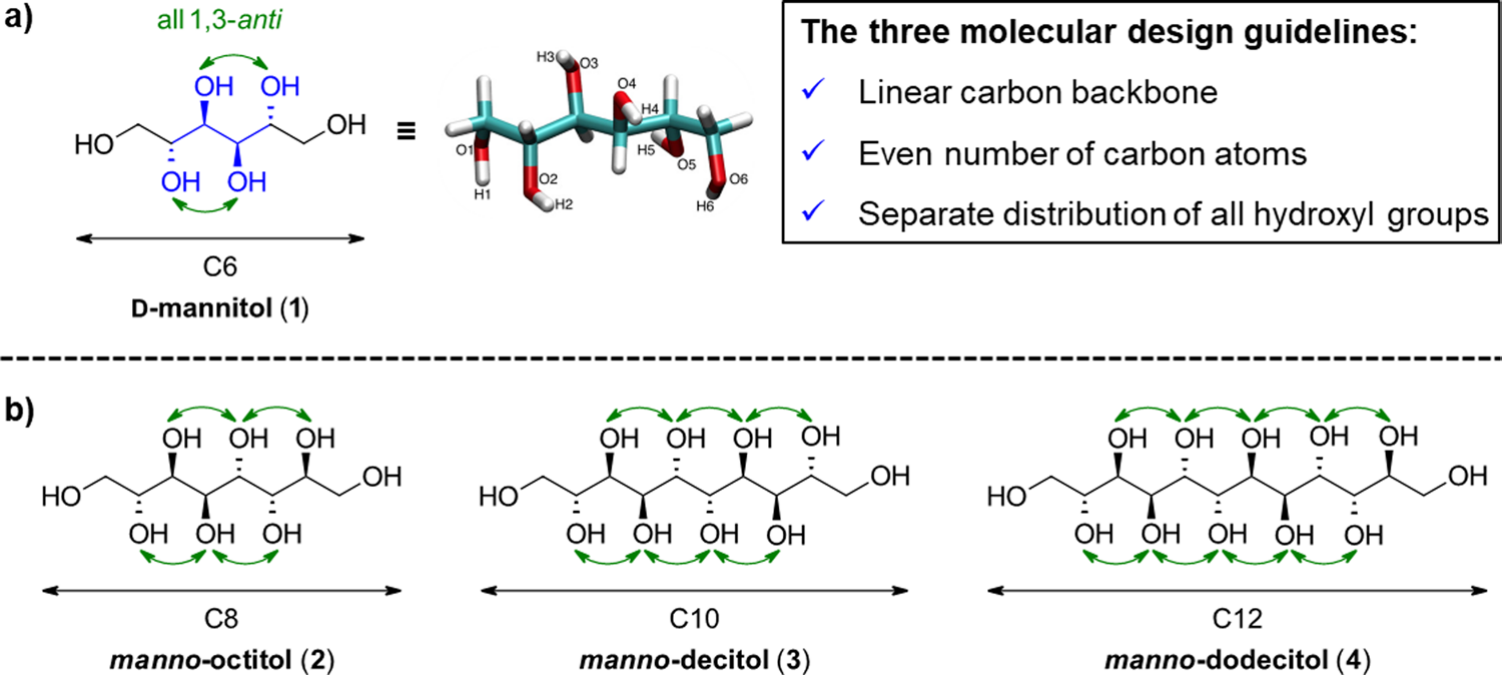
**a** The molecular structure of d-mannitol (**1**) in the solid phase obtained from MD simulations by Inagaki and Ishida [14], showing the perfect alignment due the 1,3-*anti*-distrubtion of all hydroxyl groups. **b** The designed *manno*-configured, higher-carbon sugar alcohols with up to 12 carbon atoms as larger analogues of d-mannitol (**1**)

In a recent computational study, the natural, six-carbon sugar alcohols galactitol, mannitol, sorbitol (glucitol), and iditol were examined using molecular dynamics (MD) simulation technique to demonstrate the impact of the distribution of the hydroxyl groups and therefore three-dimensional intermolecular hydrogen bond network in the solid and liquid phase [14]. Based on the findings, experimentally unknown, higher-carbon sugar alcohols with up to 20 carbon atoms in the backbone were further investigated as potential PCMs. Three guidelines for the molecular design of sugar alcohols with large thermal storage densities were proposed: (1) a linear elongation of the carbon backbone, (2) separate distribution of all hydroxyl groups, meaning a 1,3-*anti*-relationship, and (3) an even number of carbon atoms in the backbone as it can be found in d-mannitol (see Fig. 1a) [15]. Following these guidelines, sugar alcohols with more than six carbon atoms in the backbone were designed and the so-called “*manno*-configured” sugar alcohols with up to 12 carbon atoms are displayed in Fig. 1b. Additionally, two diastereomers of the *manno*-octitol (**2**) with one and two 1,3-*syn*-relationships between hydroxyl groups were investigated in the study, predicting a substantial decrease in the melting point and latent heat of fusion.

**Fig. 2:**
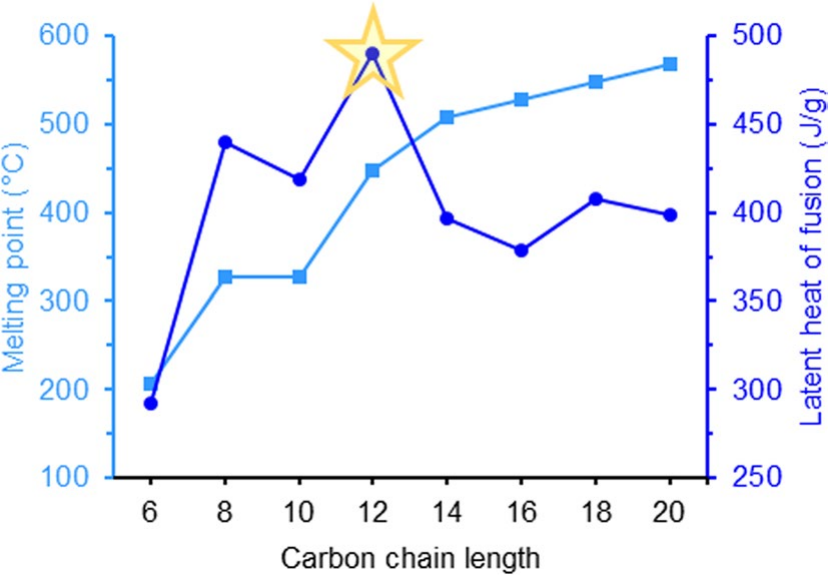
Computational predicted melting points (in °C) and thermal storage densities (in J/g) of *manno*-configured higher-carbon sugar alcohols [15]

As it can be seen in Fig. 2, for the C12 sugar alcohol, *manno*-dodecitol (**4**), the highest thermal storage density of almost 500 J/g was calculated. In general, for all investigated sugar alcohols thermal storage densities above 350 J/g are predicted, thus exceeding experimentally determined values of all organic substance explored as PCM [16]. Structures with an uneven number of carbon atoms or 1,3-*syn*-relationships between hydroxyl groups, thus violating one of the guidelines, were predicted to exhibit substantially lower heat storage capacities.

However, these higher-carbon sugar alcohols have not been experimentally investigated as PCMs yet and are neither commercially available nor readily available by synthesis. Therefore, we set out to synthesize *manno*-configured higher-carbon sugar alcohols, in order to confirm the accuracy of the MD simulations and theoretical calculations and contribute to the understanding which structural features are relevant to end up at such extraordinary energy storage characteristics. To further emphasize the rules for the structural criteria, we were also interested in an analogous C7 sugar alcohol and in the corresponding epimer, bearing one imperfect 1,3-*syn*-realtionship.

In general, higher-carbon sugar species and sugar alcohols have been synthesized in the past, especially by Brimacombe in the 1980s [17–20]. However, these syntheses are generally elaborate requiring protection strategies and are often plagued by poor stereoselectivity [21]. In the last two decades the indium-mediated acyloxyallylation (IMA) has evolved as a useful tool in the synthesis of higher-carbon sugar species with good control of stereoselectivity [22–24]. This method allows a more elegant and direct elongation of unprotected aldoses by three carbon atoms towards an enitol species by the addition of an indium organyl, in situ formed from a bromopropenyl ester and indium. In the transformation, the *lyxo*-configured product is obtained as the major product with good selectivity (> 60). The *lyxo*-configuration represents *syn*-orientation between the former O2-hydroxyl group and the hydroxyl group derived from the former aldehyde and *anti*-orientation between the two new hydroxyl groups (Scheme 1). Thus a suitable starting material is elongated in the stereochemical fashion desired for the predicted putative PCM structures.

**Figure:**
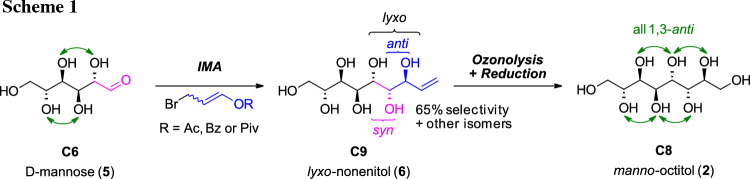

Within our recent study, the targeted *manno*-configured higher-carbon sugar alcohols were in fact found to be readily accessible via IMA and subsequent conversion of the terminal double bond via an ozonolysis and reduction protocol (see Scheme 1). Further, the C2-epimers of the sugar alcohols bearing one 1,3-*syn* relationship were obtained from the second major diastereomer formed in the IMA, the *xylo*-configured enitol (*syn*/*syn*-addition) and also studied.

With our strategy, we successfully synthesized sufficient material of first representatives of the predicted PCM structures from the higher-carbon sugar families to evaluate their thermal properties experimentally and allow comparison to and potentially refinement of the prediction model by Inagaki and Ishida [14, 15] in the future.

## Results and discussion

### Synthesis of heptitols

In addition to the sugar alcohols fulfilling all proposed criteria, we were also interested in more representatives of higher-carbon sugar alcohols and started the synthetic investigations aiming two heptitols, one with all hydroxyl-groups in an 1,3-*anti*-relationship (*manno*-heptitol **7**) and one bearing one 1,3-*syn*-relationship (*gluco*-heptitol **11**). The retrosynthetic analysis (Scheme 2) shows that the *manno*-heptitol (**7**) can be obtained from the described l-*glycero*-d-*manno*-octentitol (**8**) and the *gluco*-heptitol (**11**) from the corresponding l-*glycero*-d-*gluco*-octentiol (**12**) via ozonolysis and subsequent reduction of the obtained aldose. Up to the enitol stage, the synthesis of the C7 analogue from l-lyxose (**9**) was well established within our lab [23] and both the targeted all 1,3-*anti* product and an isomer with one 1,3-*syn* relationship were available in the lab.

**Figure:**
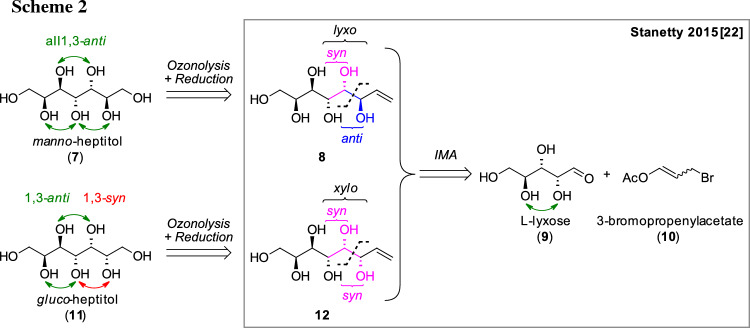

Initially, the reduction of the corresponding l-*glycero*-d-*manno*-heptose [23] was attempted using standard protocols (NaBH_4_ in an aqueous solution [25]). However, this reduction turned out to be sluggish and not as straightforward as for simpler sugars. Alternatively, the unprotected enitol **8** was submitted to ozonolysis and the ozonides were directly reduced to the corresponding alcohols by the addition of NaBH_4_. However, initial experiments with unprotected enitol **8** showed that it is beneficial to protect all free hydroxyl groups of the enitol (**13**) to allow complete and direct reduction to the sugar alcohol after the ozonolysis.

Therefore, the *manno*-enitol **8** was transformed into the peracetate **13** and the ozonolysis reaction was performed subsequently. After complete conversion of **13** to the ozonide species, NaBH_4_ was added directly to the reaction mixture to obtain the desired alcohol. However, low isolated yields were initially observed which were attributed to losses of the formed alcohol product into the aqueous phase during the work-up procedure due to the cleavage of one or more acetate groups during reduction. For complete isolation of the sugar alcohol, re-acetylation was performed directly after the ozonolysis–reduction protocol to obtain the peracetylated sugar alcohol upon simple aqueous workup. This was then immediately submitted to global deprotection under Zemplén condition giving the *manno*-heptitol (**7**) as the final product with an overall yield of 74% starting from the octenitol peracetate **13** (Scheme 3, top). The *gluco*-octenitol (**12**), available as the corresponding peracetate **14** from earlier work, was transformed into the *gluco*-heptitol (**11**) via the protocol described above and isolated in an overall yield of 73%. This sugar alcohol is a C7 epimer exhibiting one imperfect 1,3-*syn* relationship (*gluco*-configuration) compared to the ideally configured *manno*-heptitol (**7**) (Scheme 3, bottom).

**Figure:**
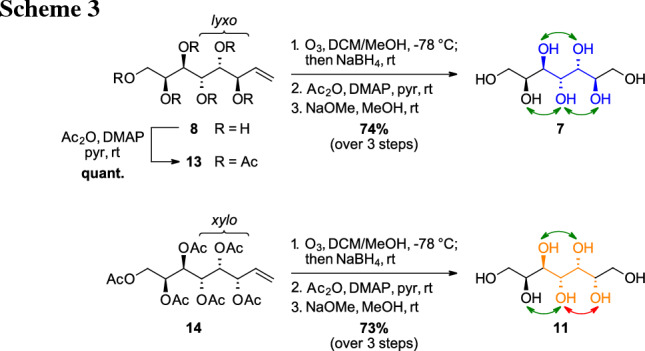

### Synthesis of octitols

The retrosynthetic analysis for the octitols (Scheme 4) showed that the *manno*-octitol (**2**) can be obtained via the developed protocol from the one-carbon longer enitol species **6**. This sugar derivative is accessible from the natural hexose d-mannose (**5**) via indium-mediated acyloxyallylation with high selectivity for the desired *lyxo*-configured product [22]. Based on our own research [23, 24], the *xylo*-configured enitol **16** is expected as the second major product in the IMA, giving access to another *gluco*-octitol (**11**) with one 1,3-*syn*-relationship en route towards **2**.

**Figure:**
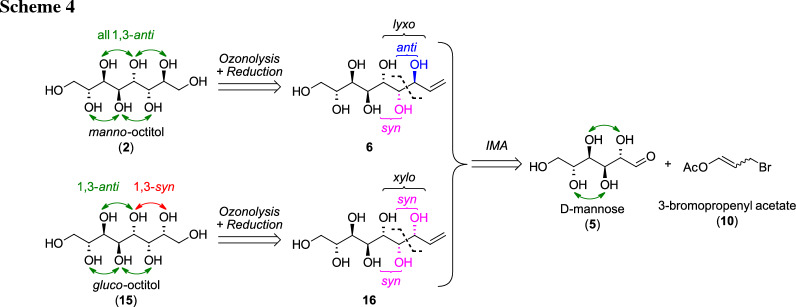

The synthesis was started from commercial d-mannose (**5**) and the IMA was first attempted under the “standard reaction conditions” where EtOH is used as solvent and 3-bromopropenyl acetate (**10**) as the elongation reagent in a Barbier-type protocol with indium [23]. However, compared to l-lyxose (**9**), d-mannose (**5**) is less soluble in EtOH and also has also a lower open-chain content (OCC, representing availability as aldehyde species) in aqueous solution of only ~ 0.03% [26]. Therefore, the reaction did not proceed to full conversion under the original conditions. For complete dissolution of the sugar, the reaction had to be performed in a very diluted solution of less than 1% (w/v) regarding the sugar starting material. Due to this, efficient stirring and quick addition of the reagent **10** and indium became even more crucial to allow fast reaction with the sugar and suppress side reactions (Wurtz-type dimerization) and hydrolysis of **10** [24, 27]. Exchange of solvent to water was not giving better result which is consistent with literature [22]. Upon peracetylation and subsequent Zemplén deacetylation a mixture of enitol diastereomers was isolated, still containing reagent-based impurities. Via integration of the H-3 signal in the ^1^H NMR the ratio of *lyxo* **6**: *xylo* **16**: *ribo* (*ribo*-isomer not shown) was determined being 65:25:10. The stereochemistry of the formed diastereomers was proposed based on the studies of Stanetty et al. [23] and Draskovits et al. [24] where this pattern was found for all investigated carbohydrates, independent of the stereochemistry at C2 and C3. The structures of the main *lyxo-*isomer **6** was confirmed by comparison to literature [22] and the fact that the corresponding sugar alcohol **2** is symmetric according to NMR-analysis (vide infra). The stereochemistry of the second putative *xylo*-configured isomer **16** is further corroborated by comparison of the ^1^H NMR spectra of nonetiols **6** and **16** with corresponding octenitols **8** and **12** [23] (see SI).

As for the l-*glycero*-d-*manno*-octentiol (**8**) [23], the *lyxo*-isomer **6** could be isolated in 33% yield over all steps from the mixture upon trituration with *i*-PrOH and recrystallization from H_2_O due to its superior crystallinity (Scheme 5, left path). The *xylo*-configured entiol **16** was obtained in pure form after acetylation of the residual enitol mixture, derived from the mother liquors of the prior trituration–recrystallization procedure, and column chromatography to give the corresponding peracetate **17** (Scheme 5, right path).

**Figure:**
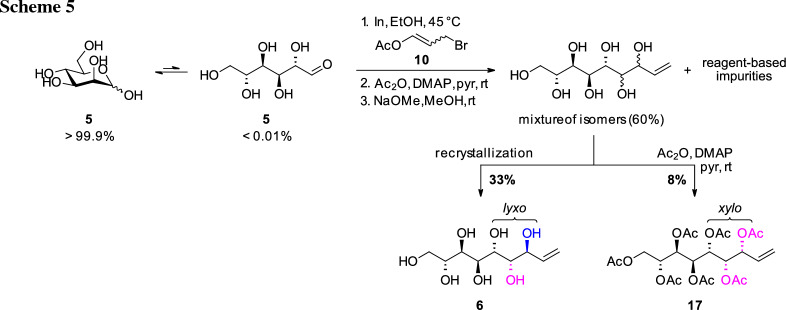

To obtain the desired *manno*-octitol (**2**), the nonenitol **6** was acetylated giving the peracetate **18** that was submitted to ozonolysis. Following the protocol as it has been described for the heptitols the sugar alcohol **2** was isolated with 56% overall yield (Scheme 6, top). The *xylo*-enitol that was directly obtained as the peracetate **17** after the IMA was transformed into the corresponding *gluco*-octitol (**15**) using the same reaction sequence with 72% yield (Scheme 6, bottom).

**Figure:**
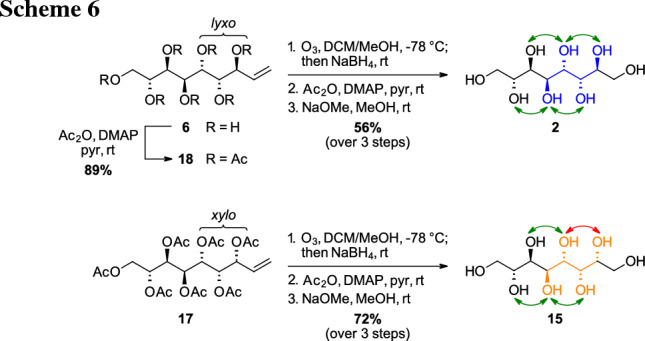

**Fig. 3:**
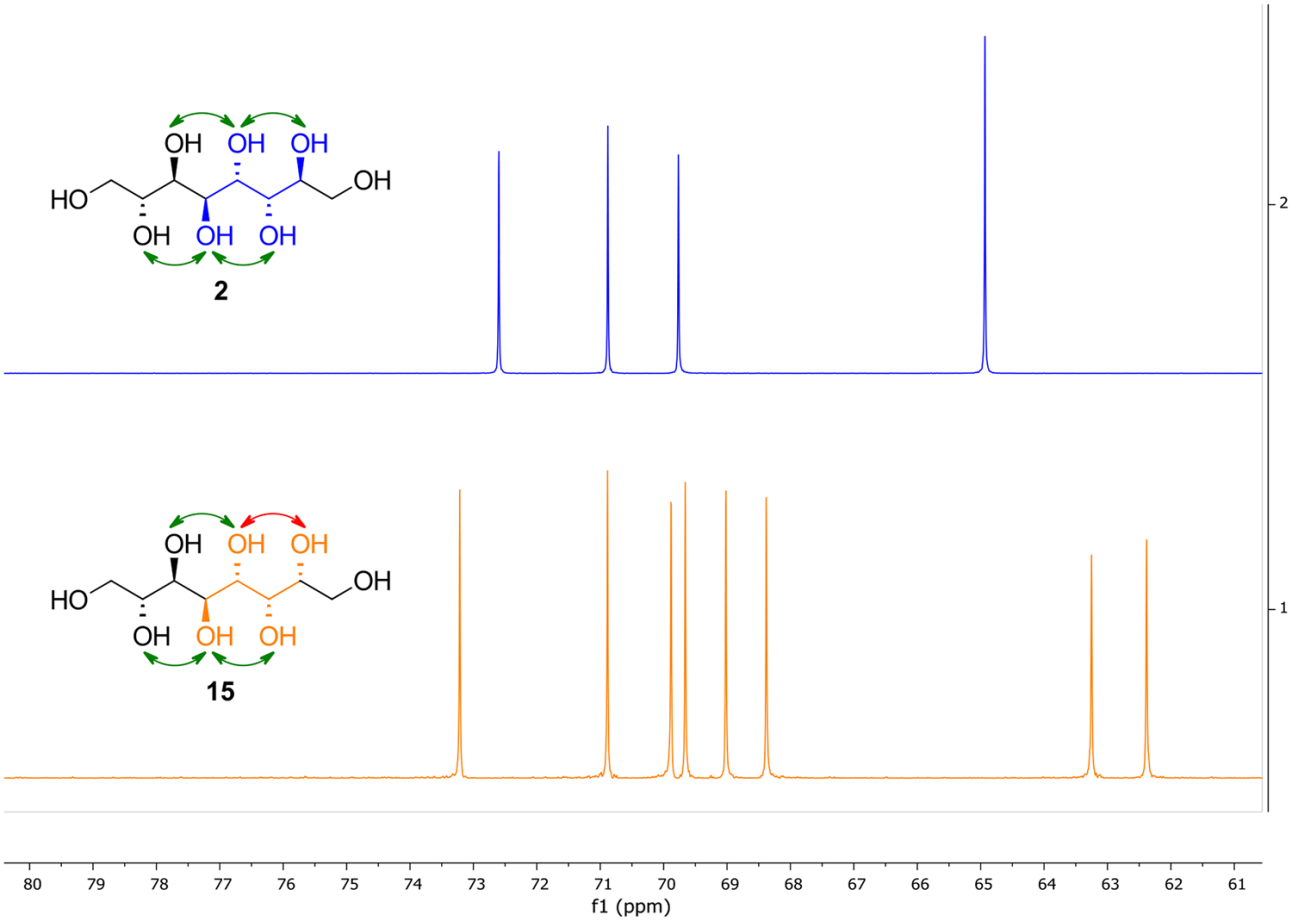
^13^C NMR spectra of *manno*-octitol** 2** (151 MHz, D_2_O, top) and *gluco*-octitol **15** (101 MHz, D_2_O, bottom) in comparison

Due to the performed reaction pathway (ozonolysis, then reduction) no change of the stereocenters compared to the enitol species is expected. The symmetry of compound **2** (*meso*-compound), observed via only four signals in the ^13^C NMR spectrum from the eight carbon atoms present in the molecule (Fig. 3, top), further corroborates the assigned stereochemistry. For the *gluco*-octitol **15**, that does not possess any symmetry, eight signals were observed (Fig. 3, bottom). Both compounds are known in literature, however, no spectral data was available so far.

Noteworthy, further elongation of the corresponding octose (obtained via ozonolysis from nonenitol **6**) towards the longer *manno*-configured sugar alcohols could not be accomplished under the reported conditions for IMA. Those interesting compounds are currently pursued via another strategy and will be reported in due time.

### Physical properties of the synthesized higher-carbon sugar alcohols

The four higher-carbon sugar alcohols were then investigated regarding their thermal properties via simultaneous thermal analysis (STA). STA combines differential scanning calorimetry (DSC) with thermogravimetric analysis (TG). From the DSC curves, the melting temperature (onset of the curve) and melting enthalpies (area of the peak) can be determined while the TG curves provide information on the thermal stability of the investigated substance or residual solvent in the sample. For the STA measurements the synthesized materials were recrystallized from H_2_O or MeOH/H_2_O mixtures (see “Experimental” section) since the degree/quality of crystallinity has a major impact on the behavior of the compound during the heating process.

**Fig. 4:**
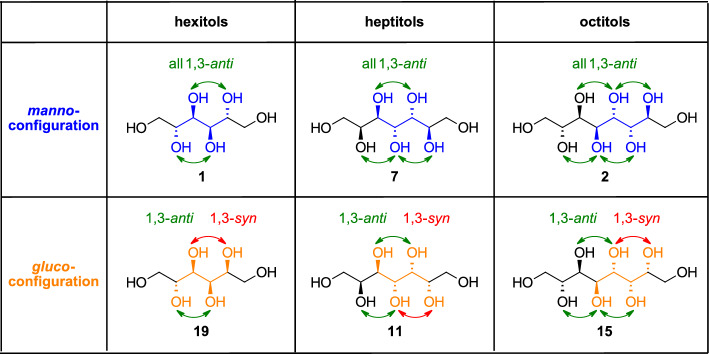
All sugar alcohols that were evaluated according to their thermal properties

**Fig. 5:**
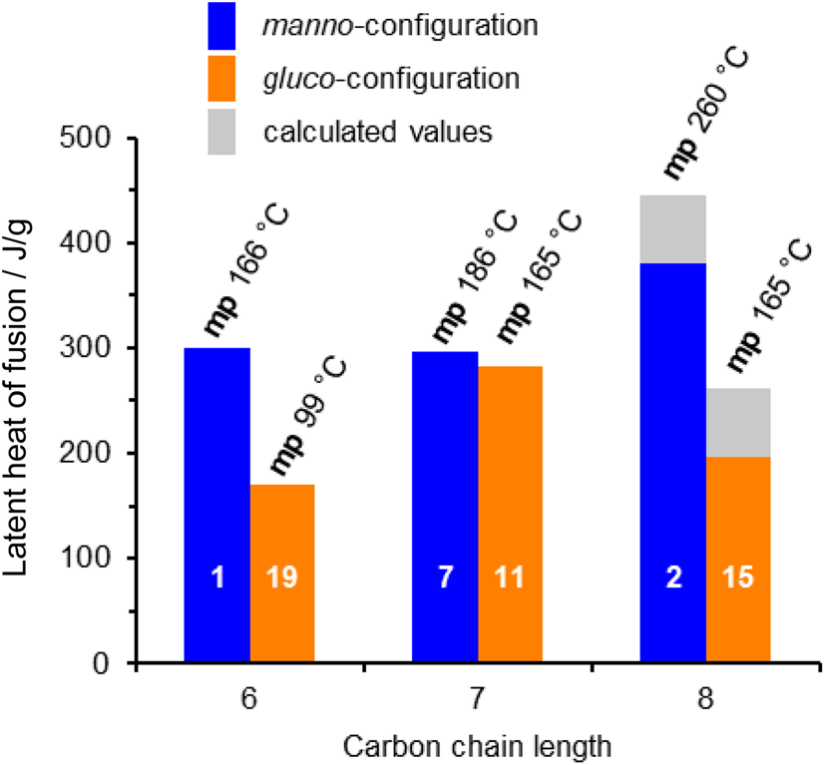
Measured latent heats of fusion (in J/g) and melting points (in °C) of the synthesized sugar alcohols compared to literature values of d-mannitol (**1**) and d-glucitol (**19**) [5]. The values for the two octitols **2** and **15** are further compared to the calculated ones by Inagaki and Ishida [15]

All sugar alcohols that were investigated are summarized and displayed in Fig. 4. For the hexitols **1** and **19**, literature values were considered in the following Fig. 5 since these natural sugar alcohols have already been intensively investigated as PCM candidates [5].

First of all, looking at the two different configurations *manno* and *gluco* the findings by Inagaki and Ishida [15] that the distribution of the hydroxyl groups has a major impact on the thermal storage density under otherwise perfect settings is clearly supported. The perfectly aligned *manno*-octitol (**2**) possesses a significantly higher latent heat of fusion (381 J/g) than its C2-epimer *gluco*-octitol (**15**) (196 J/g) bearing only one 1,3-*syn*-relationship. However, this effect does not really exist when it comes to the heptitols, where an uneven number of carbon atoms is present in the backbone, leading to comparably lower values of latent heat for both configurations (*manno* **7**: 296 J/g; *gluco* **11**: 283 J/g). Consequently, the proposed rule that an even number of carbon atoms together with the perfect distribution of all hydroxyl group is beneficial as it is also found in this first set of compounds. Within the *manno*-configured sugar alcohols (blue in Fig. 5), the heptitol **7** and hexitol **1** possess rather similar latent heats of fusion even though longer chains are usually associated with higher values. It is possible that this arises from compensation of a constructive effect of elongation by a destructive one in terms of odd/even number of carbon atoms, however only comparison within a larger number of compounds will allow an answer to this hypothesis.

Most importantly, the “perfect” higher-carbon sugar alcohol, the octitol **2,** showed the highest value of all investigated compounds and also outperforms organic PCMs that have been synthesized and evaluated as suitable candidates. Additionally, for the octitols **2** and **15** the experimental values could be compared to calculated ones (displayed as grey bars in Fig. 5). The results show that a rather precise and trustworthy prediction of the physical properties of sugar alcohols was achieved in the MD simulations by Inagaki and Ishida [15]. For both configurations the actual, measured values are slightly lower compared to the calculated ones with a difference of ~ 60 J/g (*manno* **2**: 440 (calc.) vs. 381 J/g (exp.); *gluco* **15**: 257 (calc.) vs. 196 J/g (exp.)). In general, deviations between the “real”, experimentally determined values and predicted ones might be attributed to imperfections of the synthesized material regarding residual solvent in the sample and imperfect crystallization leading to the formation of crystal cells with defects, or partial decomposition during the melting process can also occur. Nevertheless, the *manno*-octitol (**2**) still possesses an outstandingly high latent heat of fusion compared to other organic PCMs making it an interesting candidate for further investigations as a PCM.

## Conclusion

In conclusion, we have shown a short synthetic route to four higher-carbon sugar alcohols via IMA and ozonolysis with following reduction starting from two aldoses, l-lyxose (**9**) and d-mannose (**5**). The synthesized compounds were then investigated in respect to their properties as PCM candidates by measuring their melting points and latent heat of fusion via STA. The experimental values were found to be in good agreement with the predicted, calculated ones by Inagaki and Ishida [15] in their MD simulation study. The “perfect” higher-carbon sugar alcohol *manno*-octitol (**2**) that fulfills all structural criteria was found to indeed possesses an outstanding high latent heat of fusion of ~ 380 J/g with a melting point of 260 °C, compared to other organic PCMs. The syntheses have been conducted in multi 100 mg scale, providing sufficient material for their evaluation. We have shown in earlier work, that IMA can be scaled to multi 10 g scale. Following up on these findings, further non-natural sugar alcohols are currently under investigation within our group in respect to their synthesis as well as thermal properties. Next to the longer *manno*-decitol (**3**) and *manno*-dodecitol (**4**) structures another sugar alcohol family, derived from galactitol, is under investigation since galactitol also fulfills all stated criteria and possesses an even higher thermal storage density of ~ 330 J/g than d-mannitol (**1**).

## Experimental

All chemicals were purchased from commercial sources with a purity of  > 95%, unless noted different, and used without further purification. Water-free solvents were either available from commercial sources in bottles with a septum stored over molecular sieve or a PureSolv solvent purification system by Innovative Technology. When Dowex H^+^ resin was used, it was washed with the respective solvent prior to use.

Thin-layer chromatography (TLC) for reaction monitoring and fraction analysis from column chromatography was performed on silica gel 60 F_254_ plates or HPTLC-plates (silica gel 60 F_254_ with concentration zone 20 × 2.5 cm). The spots were visualized by staining the plates with anisaldehyde solution (180 cm^3^ EtOH, 10 cm^3^ anisaldehyde, 10 cm^3^ H_2_SO_4_ (conc.), 2 cm^3^ AcOH), permanganate solution (3.0 g KMnO_4_, 20.0 g K_2_CO_3_, 250 mg KOH, 300 cm^3^ H_2_O) or cerium molybdate (“Mostain”, 21.0 g (NH_4_)_6_Mo_7_O_24_∙2H_2_O, 1.0 g Ce(SO_4_), 31 cm^3^ H_2_SO_4_ (conc.), 500 cm^3^ H_2_O).

For flash column chromatography, columns were packed with silica gel from Merck with a pore size of 40–63 μm. Purification was either done by hand using standard glass columns or on a Büchi Sepacore Flash System (2 × Büchi Pump Module C-605, Büchi Pump Manager C-615, Büchi UV Photometer C-635, Büchi Fraction Collector C-660).

LC–MS analysis was performed on a Nexera X2® UHPLC system (Shimadzu®, Kyoto, Japan) comprised of LC-30AD pumps, a SIL-30AC autosampler, CTO-20AC column oven, DGU-20A5/3 degasser module. Detection was accomplished by an SPD-M20A photo diode array and a LCMS-2020 mass spectrometer. Separations were either performed using a Waters® XSelect® CSH™ C18 2.5 µm (3.0 × 50 mm) Column XP at 40 °C and a flowrate of 1.7 cm^3^/min or with a Waters® XBridge® BEH Amide 2.5 µm (3.0 × 50 mm) Column XP at 40 °C and a flowrate of 1.3 cm^3^/min. UHPLC grade water and acetonitrile containing 0.1% formic acid was used as the mobile phase.

Accurate mass analysis was performed on an Agilent 6230 AJS ESI-TOF mass spectrometer with ESI ionization method or Q Exactive Focus, ESI, FIA injection, mobile phase 18% MeCN with 0.1% formic acid.

^1^H NMR and ^13^C NMR spectra were recorded at ambient temperature (25 °C) in the solvent indicated using a Bruker Avance UltraShield 400 MHz and an Avance III HD 600 MHz spectrometer. Processing of the data was performed with standard software and all spectra were calibrated to the solvent residual peak [28]. Chemical shifts (*δ*) are reported in ppm, coupling constants (*J*) in hertz (Hz) and multiplicities are assigned as s = singlet, d = doublet, t = triplet, q = quartet, m = multiplet, bs = broad singlet etc. All assignments are based on 2D-sepctra (COSY, phase sensitive HSQC, HMBC—depending on the molecule).

If not stated different, melting points were recorded with a Kofler-type Leica Galen III micro hot stage microscope and a BÜCHI Melting Point B 545 with a 40%/90% threshold and a heating rate of 1.0 °C/min.

Specific rotations were measured on an Anton Paar MCP 500 at the specified conditions. If no value is stated, the corresponding substance showed insufficient solubility in a suitable solvent.

Ozone enriched oxygen was generated using a Triogen LAB2B Ozone generator.

Simultaneous thermal analysis (STA) including differential scanning calorimetry (DSC) and thermogravimetric analysis (TG) measurements were performed on a Netzsch STA 449 F1 Jupiter under nitrogen atmosphere with a heating and cooling rate of 10 K/min if not stated otherwise. Samples were measured using Al pans with a hole in the lid. Latent heats of fusion were determined by linear integration of the peak appearing in the DSC curve, melting points from the extrapolated onset.

**Fig. 6:**
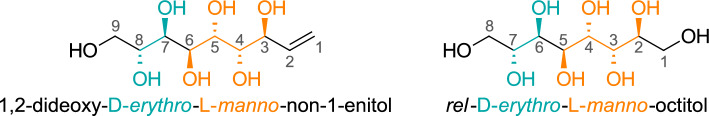
Exemplary structures for numbering of compounds

Compounds were named according to IUPAC systematic standards, in general. When it comes to higher-carbon sugar species (more than six carbon atoms), names were generated by dividing the sugar species into groups of up to four chiral centers consequently starting from the chiral center next to the former reducing end (on the right for all displayed structures). To these groups, configurational prefixes were assigned, and the name was built up by putting the prefix of the group that is farthest from the right end (C1) first. This group may contain less than four carbon atoms. Numbering of compounds was performed in the same way, always starting with 1 at the former reducing end as shown in the exemplary structures in Fig. 6.

### 3,4,5,6,7,8-Hexa-*O*-acetyl-1,2-dideoxy-l-*glycero*-d-*manno*-oct-1-enitol (13, C_20_H_28_O_12_)

The synthesis and isolation of l-*glycero*-d-*manno*-oct-1-enitol (**8**) is already described elsewhere [23]. The octenitol **8** (200 mg, 0.96 mmol, 1.00 eq.) was taken up in pyridine (8.0 cm^3^) and Ac_2_O (1.63 cm^3^, 17.3 mmol, 18.0 eq.) was added dropwise under ice-bath cooling. After a homogeneous solution was formed, a spatula tip of DMAP was added and the reaction mixture was stirred at rt until TLC (CHCl_3_/MeOH/H_2_O 14:7:1, LP/EtOAc 1:1) indicated complete conversion to a very non-polar component. Excessive reagent was quenched by the slow addition of MeOH (0.7 cm^3^) under ice-bath cooling. The reaction mixture was diluted with DCM (100 cm^3^) and extracted with 1 N HCl until the aq. phase remained acidic. The combined aq. phase was extracted with DCM once (10 cm^3^) and the pooled organics were washed with sat. aq. NaHCO_3_ and brine. After drying over MgSO_4_ the solvent was evaporated giving the octenitol peracetate **13** as a colorless solid (440 mg, quant.). No further purification was necessary at this stage. M.p.: 127.6–127.8 °C (DCM); ^1^H NMR (400 MHz, CDCl_3_): *δ* = 5.69 (ddd, *J* = 17.2, 10.3, 7.8 Hz, 1H, H-2), 5.51 (dd, *J* = 10.0, 1.9 Hz, 1H, H-5), 5.37–5.31 (m, 1H, H-1a), 5.28 (dt, *J* = 10.3, 2.4 Hz, 2H, H-6, H-1b), 5.23 (dd, *J* = 8.2, 1.9 Hz, 1H, H-4), 5.21–5.17 (m, 1H, H-7), 5.13 (t, *J* = 8.0 Hz, 1H, H-3), 4.28 (dd, *J* = 11.7, 4.9 Hz, 1H, H-8a), 3.82 (dd, *J* = 11.7, 7.3 Hz, 1H, H-8b), 2.11 (s, 3H, C(= O)C*H*_3_), 2.09 (s, 3H, C(=O)C*H*_3_), 2.06 (s, 3H, C(=O)C*H*_3_), 2.05 (s, 3H, C(=O)C*H*_3_), 2.03 (s, 3H, C(=O)C*H*_3_), 2.01 (s, 3H, C(=O)C*H*_3_) ppm; ^13^C NMR (101 MHz, CDCl_3_): *δ* = 170.6, 170.4, 170.2, 170.0, 169.72, 169.69 (6 × *C*(=O)CH_3_), 132.4 (C2), 121.2 (C1), 72.1 (C3), 69.4 (C4), 67.7 (C6, C7), 66.5 (C5), 62.4 (C8), 21.2, 21.0, 20.9, 20.79, 20.76 (6 × C(=O)*C*H_3_) ppm; HR-MS (^+^ESI-TOF): *m*/*z* calc. for C_20_H_28_NaO_12_ ([M + Na]^+^) 483.1478, found 483.1481.

### l-*Glycero*-d-*manno*-heptitol = Perseitol (**7**)

*Step 1—Ozonolysis and reduction*: Octenitol peracetate **13** (440 mg, 0.956 mmol, 1.00 eq.) was dissolved in DCM/MeOH (3:1, dry, 44 cm^3^) and the solution was chilled to −78 °C using a liquid N_2_/acetone bath. Then, ozone was bubbled through until the solution turned dark blue. The reaction mixture was stirred for 30 min at −78 °C, then oxygen was bubbled through until the blue color diminished. TLC analysis (LP/EtOAc 1:1) was performed showing complete conversion of the starting material. Then, NaBH_4_ (108 mg, 2.87 mmol, 3.00 eq.) was added and stirring was continued at rt to reduce the formed ozonides until TLC analysis and LC–MS confirmed the absence of any intermediary formed aldehyde. Then, AcOH (7 cm^3^) was added, and the reaction mixture was evaporated in vacuo giving a colorless crude residue.

*Step 2—Acetylation*: The residue was taken up pyridine (2.5 cm^3^) and Ac_2_O (0.27 cm^3^, 2.9 mmol, 3.0 eq.) was added under ice-bath cooling. After a homogeneous solution was formed, a spatula tip of DMAP was added and the reaction mixture was stirred at rt for 18 h. Complete conversion to the peracetate was confirmed via LC–MS. Excessive reagent was quenched by the addition of MeOH (0.25 cm^3^) under ice-bath cooling. The reaction mixture was diluted with EtOAc (20 cm^3^) and extracted with 1 N HCl until the aq. phase remained acidic. The combined aq. phases were extracted with EtOAc once (10 cm^3^) and the pooled organic phases were washed with sat. aq. NaHCO_3_ and brine. After drying over MgSO_4_ the solvent was evaporated giving the 1,2,3,4,5,6,7-hepta-*O*-acetyl**-**l-*glycero*-d-*manno*-heptitol (**SI-1**) (480 mg) that was used in the next step without further purification. For analysis, an analytical sample was purified by column chromatography (LP/EtOAc 3:1). Analytical data for intermediate **SI-1** (peracetate): ^1^H NMR (400 MHz, CDCl_3_): *δ* = 5.49 (dd, *J* = 10.0, 1.9 Hz, 1H), 5.35 (dd, *J* = 8.9, 1.9 Hz, 1H), 5.24 (dd, *J* = 10.0, 2.0 Hz, 1H), 5.16 (ddd, *J* = 7.0, 4.9, 2.0 Hz, 1H), 5.00 (dq, *J* = 8.8, 2.8 Hz, 1H), 4.28 (dd, *J* = 11.7, 4.9 Hz, 1H), 4.21 (dd, *J* = 12.5, 2.8 Hz, 1H), 4.02 (dd, *J* = 12.5, 5.2 Hz, 1H), 3.81 (dd, *J* = 11.7, 7.2 Hz, 1H), 2.11 (s, 3H), 2.09 (s, 3H), 2.08 (s, 3H), 2.06 (s, 6H), 2.04 (s, 3H), 2.01 (s, 3H) ppm; ^13^C NMR (101 MHz, CDCl_3_): *δ* = 170.7, 170.6, 170.4, 170.1 (2 ×), 170.0, 169.8 (7 × *C*(=O)CH_3_), 68.2, 67.8, 67.7, 67.4, 66.7 (5 × CH), 62.4 (C1/C7), 62.0 (C1/C7), 21.1, 20.9, 20.83, 20.81 (2 ×), 20.80, 20.7 (7 × C(=O)*C*H_3_) ppm; HR-MS (^+^ESI-TOF): *m*/z calc. for C_21_H_30_NaO_14_ ([M + Na]^+^) 529.1533, found 529.1547. Spectral data is in accordance with literature [29].

*Step 3—Deacetylation*: The peracetate **SI-1** (480 mg, 0.948 mmol, 1.00 eq.) was taken up in MeOH (5 cm^3^) and NaOMe (30% in MeOH, 10 mm^3^, pH ~ 8) was added at rt under stirring. After 30 min, reaction monitoring via LC–MS indicated complete conversion to the desired sugar alcohol. The reaction mixture was neutralized by the addition of Dowex-H^+^ and water was added to dissolve the formed heptitol. The resin was filtered off and the filter cake washed with additional water. The filtrate was evaporated giving the *manno*-heptitol **7** as a colorless solid (150 mg, 74% yield over 3 steps), pure according to NMR. Analytical data for **7**: m.p.: 184–186 °C (MeOH/H_2_O), 186.0 °C (MeOH/H_2_O, from DSC) (Ref. [18] 186–187 °C (aq. EtOH)); [*α*]_D_^20^ =  + 7.2° cm^2^ g^−1^ (*c* = 1.0, H_2_O); ^1^H NMR (400 MHz, D_2_O): *δ* = 4.04–3.96 (m, 1H), 3.93 (d, *J* = 9.5 Hz, 1H), 3.88 (dd, *J* = 11.7, 2.6 Hz, 1H), 3.86–3.73 (m, 2H), 3.74–3.64 (m, 4H) ppm; ^13^C NMR (101 MHz, D_2_O): *δ* = 71.0, 70.3, 69.3, 69.2, 68.3, 63.4, 63.3 ppm. Spectral data is in accordance with literature [30].

*Purification for DSC:* The product was recrystallized from MeOH/H_2_O (4:1, 10 cm^3^) with slow cooling rate to allow a better growth of crystals that can be further analyzed in STA measurements.

### l-*Glycero*-d-*gluco-*heptitol (**11**)

The synthesis and isolation of l-*glycero*-d-*gluco*-oct-1-enitol hexaacetate (**14**) is already described elsewhere [23].

*Step 1—Ozonolysis and reduction*: The octenitol peracetate **14** (520 mg, 1.13 mmol, 1.00 eq.) was dissolved in DCM/MeOH (3:1, dry, 44 cm^3^) and the solution was chilled to −78 °C using a liquid N_2_/acetone bath. Then, ozone was bubbled through until the solution turned dark blue. The reaction mixture was stirred for 30 min at −78 °C, then oxygen was bubbled through until the blue color diminished. TLC analysis (LP/EtOAc 1:1) was performed showing complete conversion of the starting material. Then, NaBH_4_ (128 mg, 3.39 mmol, 3.00 eq.) was added and stirring was continued at rt to reduce the formed ozonides until TLC analysis and LC–MS confirmed the absence of any intermediary formed aldehyde. Then, AcOH (7 cm^3^) was added, and the reaction mixture was evaporated in vacuo giving a colorless crude residue.

*Step 2—Acetylation*: The residue was taken up pyridine (2.5 cm^3^) and Ac_2_O (0.32 cm^3^, 3.4 mmol, 3.0 eq.) was added under ice-bath cooling. After a homogeneous solution was formed, a spatula tip of DMAP was added and the reaction mixture was stirred at rt for 18 h. Complete conversion to the peracetate was confirmed via LC–MS. Excessive reagent was quenched by the addition of MeOH (0.2 cm^3^) under ice-bath cooling. The reaction mixture was diluted with EtOAc (20 cm^3^) and extracted with 1 N HCl until the aq. phase remained acidic. The combined aq. phases were extracted with EtOAc once (10 cm^3^) and the pooled organic phases were washed with sat. aq. NaHCO_3_ and brine. After drying over MgSO_4_ the solvent was evaporated giving the 1,2,3,4,5,6,7-hepta-*O*-acetyl**-**l-*glycero*-d-*gluco*-heptitol (**SI-2**) (565 mg) that was used in the next step without further purification. Analytical data for intermediate **SI-2** (peracetate): ^1^H NMR (400 MHz, CDCl_3_): *δ* = 5.40–5.34 (m, 2H), 5.23 (dd, *J* = 8.4, 3.4 Hz, 1H), 5.12 (dt, *J* = 7.5, 3.3 Hz, 1H), 5.04 (ddd, *J* = 8.3, 5.2, 2.8 Hz, 1H), 4.32 (dd, *J* = 12.1, 3.2 Hz, 1H), 4.23–4.15 (m, 2H), 4.03 (dd, *J* = 12.5, 5.2 Hz, 1H), 2.12 (s, 3H), 2.07–2.02 (m, 18H) ppm. Spectral data is in accordance with literature [31].

*Step 3—Deacetylation*: The peracetate **SI-2** (565 mg, 1.12 mmol, 1.00 eq.) was taken up in MeOH (5 cm^3^) and NaOMe (30% in MeOH, 10 mm^3^, pH ~ 9) was added at rt under stirring. After 30 min, reaction monitoring via LC–MS indicated complete conversion to the desired sugar alcohol. The reaction mixture was neutralized by the addition of Dowex-H^+^ and water was added to dissolve the formed heptitol. The resin was filtered off and the filter cake washed with additional water. The filtrate was evaporated giving the *gluco*-heptitol **11** as a colorless solid (174 mg, 73% yield over 3 steps), pure according to NMR. Analytical data for **11**: m.p.: 164.6 °C (MeOH/H_2_O, from DSC) (Ref. [18] 141–142 °C (aq. EtOH)); [*α*]_D_^20^ =  + 4.0° cm^2^ g^−1^ (*c* = 1.0, H_2_O); ^1^H NMR (400 MHz, D_2_O): *δ* = 3.95 (ddd, *J* = 7.7, 4.8, 3.1 Hz, 1H), 3.91 (dd, *J* = 8.5, 1.0 Hz, 1H), 3.91–3.78 (m, 4H), 3.77 (dd, *J* = 6.0, 2.7 Hz, 1H), 3.75–3.67 (m, 1H), 3.70–3.63 (m, 1H) ppm; ^13^C NMR (101 MHz, D_2_O): *δ* = 72.9, 71.5, 70.8, 69.6, 69.5, 63.2, 62.0 ppm; HR-MS (^+^ESI-TOF): *m*/*z* calc. for C_7_H_16_NaO_7_ ([M + Na]^+^) 235.0788, found 235.0801.

*Purification for DSC*: The product was recrystallized from MeOH/H_2_O (4:1, 10 cm^3^) with slow cooling rate to allow a better growth of crystals that can be further analyzed in STA measurements.

### 3-Bromoprop-1-en-1-yl acetate (**10**, mixture of (*E*)- and (*Z*)-isomer)

According to a literature protocol [23], acrolein (90%, 7.93 cm^3^, 107 mmol, 1.00 eq.) was dissolved in dry DCM (85 cm^3^) and cooled to − 20 °C using an liquid N_2_/acetone cooling bath. First, acetyl bromide (7.35 cm^3^, 101 mmol, 0.95 eq.) was added to the solution under stirring followed by anhydrous ZnCl_2_ (0.15 g, 1.1 mmol, 1.0 mol%). The reaction mixture was allowed to warm up to −15 °C by lowering the cooling bath as the exothermic reaction started and a temperature jump to + 10 °C was observed. The flask was then re-immersed in the cooling bath, and the reaction mixture was stirred at − 20 °C for 1 h. Reaction monitoring via ^1^H NMR analysis (micro work-up of a sample with Et_2_O and aq. sat. NaHCO_3_, drying over Na_2_SO_4_) confirmed full conversion of acrolein to the targeted product and the reaction mixture was poured onto a water/ice mixture (50 cm^3^). Layers were separated and the organic phase was washed with water (50 cm^3^) followed by sat. aq. NaHCO_3_ (2 × 50 cm^3^—until pH remained basic) and brine. The organic phase was dried over anhydrous MgSO_4_ and evaporated, giving the crude product (17.1 g) as a brown, oily liquid. The pure product **10** was obtained by distillation in vacuo (b.p.: 69–75 °C, 30 mbar) as a colorless to slightly yellow liquid (11.5 g, 60%) in a ratio of *E*/*Z* = 1:1.6 (according to ^1^H NMR). Spectral data is in accordance with literature [23].

### 1,2-Dideoxy-d-erythro-l-*manno*-non-1-enitol (**6**)

*Step 1—IMA:* Commercial d-mannose (**5**) (4.68 g, 26.0 mmol, 1.00 equiv.) was dissolved in dry EtOH (ca. 1 w/v%, 500 cm^3^) and the solution was heated to 45 °C. Under vigorous stirring, first indium (4.95 g, 42.1 mmol, 1.66 eq.) followed by bromopropenyl ester **10** (6.14 cm^3^, 64.9 mmol, 2.50 eq.) were added. The reaction progress was monitored via TLC (CHCl_3_/MeOH/H_2_O 14:7:1) indicating complete conversion of the sugar after 15 min. The reaction mixture was allowed to cool down to rt, then unreacted indium was filtered off, and the filtrate was evaporated to dryness giving a colorless residue (~ 24 g). 

*Step 2—Acetylation*: The residue was taken up in pyridine (180 cm^3^) and Ac_2_O (44.2 cm^3^, 468 mmol, 18.0 eq.) was added via a dropping funnel under ice-bath cooling. The reaction mixture was stirred until a solution was formed, then a spatula of DMAP was added. Stirring was continued at rt until TLC analysis (CHCl_3_/MeOH/H_2_O 14:7:1) indicated conversion to a very nonpolar species. Excessive reagent was quenched by the slow addition of MeOH (20 cm^3^) under ice-bath cooling. First, DCM (100 cm^3^) was added and extraction with cold 1 N HCl was performed until the aq. phase remained acidic. The HCl phase was extracted with DCM once and the combined org. phases were washed with sat. aq. NaHCO_3_ and brine. After drying over anhydrous MgSO_4_, the solution was evaporated to dryness giving a brown oil. 

*Step 3—Deacetylation*: The brown material was taken up in MeOH (130 cm^3^) and NaOMe (30% in MeOH, 180 mm^3^, pH ~ 8) was added under stirring at rt. Since TLC analysis (CHCl_3_/MeOH/H_2_O 14:7:1, LP/Et_2_O 1:2) after 30 min did not show complete conversion, further NaOMe (30% in MeOH, 70 mm^3^) was added. The reaction mixture was stirred for another hour not showing any further reaction progress. Neutralization was performed by the addition of Dowex-H^+^. To dissolve any precipitated enitols, water (100 cm^3^) was added and the solution was filtered. The filter cake was washed with addition water and the filtrate was evaporated to dryness giving a beige solid matter that was a mixture of diastereomers (*lyxo* **6**: *xylo* **16**: *ribo* 65: 25: 10, determined via integration of H-3 signal in ^1^H NMR) together with reagent-based impurities. The residue was triturated with *i*-PrOH (55 cm^3^) and the remaining solid collected via filtration. This was then recrystallized from water (7.5 cm^3^) to obtain a first fraction of the *lyxo*-nonenitol **6** as a colorless solid (907 mg, > 98% *lyxo*-isomer, 15% yield). The recrystallization step was repeated twice giving another crop (804 mg, > 90% *lyxo*-isomer, 13% yield). M.p.: 205.0–205.6 °C (H_2_O) (Ref. [22] 199–201 °C (H_2_O)); *R*_f_ = 0.23 (CHCl_3_/MeOH/H_2_O 14:7:1); [*α*]_D_^20^ =  − 8.6° cm^2^ g^−1^ (*c* = 1.0, H_2_O) (Ref. [22] [*α*]_D_^22^ =  − 10.0° cm^2^ g^−1^ (*c* = 2.1, H_2_O)); ^1^H NMR (400 MHz, D_2_O): *δ* = 6.03 (ddd, *J* = 17.3, 10.4, 6.9 Hz, 1H, H-2), 5.39 (d, *J* = 17.2 Hz, 1H, H-1a), 5.32 (d, *J* = 10.5 Hz, 1H, H-1b), 4.21 (t, *J* = 7.4 Hz, 1H, H-3), 3.94 (bs, 2H, H-5, H-6/H-7), 3.89 (dd, *J* = 11.7, 2.6 Hz, 1H, H-9a), 3.85 (d, *J* = 8.8 Hz, 1H, H-6/H-7), 3.81–3.75 (m, 2H, H-4, H-8), 3.69 (dd, *J* = 11.7, 6.1 Hz, 1H, H-9b) ppm; ^13^C NMR (101 MHz, D_2_O): *δ* = 137.7 (C-2), 117.7 (C-1), 72.5 (C-3), 71.6, 71.0 (C-4, C-8), 69.3, 68.3 (2 ×) (C-5/C-6/C-7), 63.3 (C-9) ppm; HR-MS (^+^ESI-TOF): *m*/*z* calc. for C_9_H_18_NaO_7_ ([M + Na]^+^) 261.0945, found 261.0940. Spectral data is in accordance with literature [22].

### 3,4,5,6,7,8,9-Hepta-*O*-acetyl-1,2-dideoxy-d-*eryhtro*-l-*manno*-non-1-enitol (**18**)

The *manno*-nonenitol **6** (450 mg, 1.89 mmol, 1.00 eq.) was taken up in pyridine (2 cm^3^) and treated with Ac_2_O (3.75 cm^3^, 39.7 mmol, 21.0 eq.) under ice-bath cooling. The reaction mixture was stirred until a homogeneous solution was obtained, then a spatula tip of DMAP was added and stirring was continued at rt for 18 h. Complete conversion to a very nonpolar spot was confirmed by TLC (CHCl_3_/MeOH/H_2_O 14:7:1). Excessive reagent was quenched by the slow addition of MeOH (1.6 cm^3^) under ice-bath cooling. After stirring for 20 min at rt, the reaction mixture was diluted with DCM (50 cm^3^) and extracted with 1 N HCl until the aq. phase remained acidic. The combined aq. phases were extracted with DCM once (10 cm^3^) and the combined organics washed with sat. aq. NaHCO_3_ and brine. After drying over MgSO_4_ the solvent was evaporated to dryness giving the desired product **18** as a colorless solid (890 mg, 89%). No further purification was necessary at this stage. M.p.: 128.4–128.6 °C (DCM) (Ref. [22] 127–128 °C (EtOAc/hexane); *R*_f_ = 0.44 (LP/Et_2_O 1:2); [*α*]_D_^20^ =  − 1.3° cm^2^ g^−1^ (*c* = 1.0, CHCl_3_) (Ref. [22] [*α*]_D_^22^ =  + 0.9° cm^2^ g^−1^ (*c* = 2.0, CHCl_3_)); ^1^H NMR (400 MHz, CDCl_3_): *δ* = 5.78–5.61 (m, 1H, H-2), 5.42 (bs, 2H, 2 × CH), 5.35–5.30 (m, 1H, H-1a), 5.29–5.23 (m, 2H, CH, H-1b), 5.11 (d, *J* = 4.1 Hz, 2H, 2 × CH), 4.99 (ddd, *J* = 8.5, 5.6, 3.0 Hz, 1H, H-8), 4.22 (dd, *J* = 12.4, 3.0 Hz, 1H, H-9a), 4.00 (dd, *J* = 12.5, 5.6 Hz, 1H, H-9b), 2.09 (s, 3H, C(=O)C*H*_3_), 2.07 (s, 6H, 2 × C(=O)C*H*_3_), 2.05 (s, 3H, C(=O)C*H*_3_), 2.04 (s, 3H, C(=O)C*H*_3_), 2.03 (s, 6H, 2 × C(= O)C*H*_3_) ppm; ^13^C NMR (101 MHz, CDCl_3_): *δ* = 170.5, 170.1, 170.0, 169.9, 169.7, 169.64, 169.59 (7 × *C*(=O)CH_3_), 132.2 (C-2), 121.1 (C-1), 72.4, 69.4, 68.3, 67.5, 66.8, 66.6 (6 × CH), 61.9 (C-9), 21.1, 20.93, 20.91, 20.75, 20.74, 20.69, 20.67 (7 × C(= O)*C*H_3_) ppm; HR-MS (^+^ESI-TOF): *m*/*z* calc. for C_23_H_32_NaO_14_ ([M + Na]^+^) 555.1684, found 555.1688. Spectral data is in accordance with literature [22].

### *meso*-d-*Erythro*-l-*manno-*octitol (2, C_8_H_18_O_8_)

*Step 1—Ozonolysis and reduction*: The *manno*-nonenitol peracetate **18** (820 mg, 1.54 mmol, 1.00 eq.) was dissolved in DCM/MeOH (3:1, dry, 70 cm^3^) and the solution chilled to −78 °C using a liquid N_2_/acetone bath. Then, ozone was bubbled through until the solution turned dark blue. The reaction mixture was stirred for 30 min at −78 °C, then oxygen was bubbled through until the blue color diminished. TLC analysis (LP/Et_2_O 1:7) was performed showing complete conversion of the starting material. NaBH_4_ (175 mg, 4.62 mmol, 3.00 eq.) was added and stirring was continued at rt to reduce the formed ozonides. After 30 min, TLC analysis confirmed the absence of any intermediary formed aldehyde, AcOH (14 cm^3^) was added, and the reaction mixture was evaporated in vacuo giving a colorless crude residue (2.3 g).

*Step 2—Acetylation*: The residue (2.3 g) was taken up pyridine (7.0 cm^3^) and Ac_2_O (0.87 cm^3^, 9.2 mmol, 6.0 eq.) was added under ice-bath cooling. After a homogeneous solution was formed, a spatula tip of DMAP was added and the reaction mixture was stirred at rt for 18 h. Complete conversion to the peracetate was confirmed via LC–MS. Excessive reagent was quenched by the addition of MeOH (0.4 cm^3^) under ice-bath cooling. The reaction mixture was diluted with DCM (100 cm^3^) and extracted with 1 N HCl until the aq. phase remained acidic. The combined aq. phases were extracted with DCM once (10 cm^3^) and the pooled organic phases were washed with sat. aq. NaHCO_3_ and brine. After drying over MgSO_4_ the solvent was evaporated giving the *meso*-1,2,3,4,5,6,7,8-octa-*O*-acetyl-d-*erythro*-l-*manno*-octitol (**SI-3**) (740 mg) that was used in the next step without further purification. Analytical data for intermediate **SI-3** (peracetate): ^1^H NMR (400 MHz, CDCl_3_): *δ* = 5.40 (bs, 2H, H-4, H-5), 5.22 (d, *J* = 8.2 Hz, 2H, H-3, H-6), 4.99 (ddd, *J* = 8.4, 5.6, 3.0 Hz, 2H, H-2, H-7), 4.23 (dd, *J* = 12.4, 3.1 Hz, 2H, H-1a, H-8a), 4.00 (dd, *J* = 12.5, 5.6 Hz, 2H, H-1b, H-8b), 2.09 (s, 6H, 2 × C(=O)C*H*_3_), 2.08 (s, 6H, 2 × C(=O)C*H*_3_), 2.06 (s, 6H, 2 × C(=O)C*H*_3_), 2.03 (s, 6H, 2 × C(=O)C*H*_3_) ppm.

*Step 3—Deacetylation*: The peracetate **SI-3** (740 mg, 1.28 mmol, 1.00 eq.) was taken up in MeOH (8 cm^3^) and NaOMe (30% in MeOH, 20 mm^3^, pH ~ 9) was added dropwise at rt under stirring. After 30 min, reaction monitoring via LC–MS indicated complete conversion to the desired sugar alcohol. The reaction mixture was neutralized by the addition of Dowex-H^+^ and water was added to dissolve the formed octitol. The resin was filtered off and the filter cake washed with additional water. The filtrate was lyophilized to obtain the *manno*-octitol **2** as a colorless solid (210 mg, 56% yield over 3 steps), pure according to NMR. Analytical data for **2**: m.p.: 262.0–262.7 °C (H_2_O); ^1^H NMR (600 MHz, D_2_O): *δ* = 3.89 (bs, 2H, H-4, H-5), 3.85 (dd, *J* = 11.8, 2.8 Hz, 2H, H-1a, H-8a), 3.81 (d, *J* = 8.9 Hz, 2H, H-3, H-6), 3.74 (ddd, *J* = 9.0, 6.3, 2.8 Hz, 2H, H-2, H-7), 3.65 (dd, *J* = 11.8, 6.3 Hz, 2H, H-1b, H-8b) ppm; ^13^C NMR (151 MHz, D_2_O): *δ* = 72.6 (C-4, C-5), 70.9 (C-2, C-7), 69.8 (C-3, C-6), 64.9 (C-1, C-8) ppm; HR-MS (^+^ESI-TOF): *m*/*z* calc. for C_8_H_18_NaO_8_ ([M + Na]^+^) 265.0894, found 265.0898.

*Purification for DSC*: The product was recrystallized from H_2_O with slow cooling rate to allow a better growth of crystals that can be further analyzed in STA measurements.

### 3,4,5,6,7,8,9-Hepta-*O*-acetyl-1,2-dideoxy-d-*erythro*-l-*gluco*-non-1-enitol (**17**, C_23_H_32_O_14_)

From the isolation process of d-*erythro*-l-*manno*-nonenitol (**6**) described earlier, the *i*-PrOH phase from the trituration and all mother liquors from the recrystallization (H_2_O phase) were combined and evaporated to dryness. The residue (2.14 g, 8.98 mmol, 1.00 eq.) was taken up in pyridine (20 cm^3^) and Ac_2_O (17.8 cm^3^, 189 mmol, 21 eq.) was added under ice-bath cooling. When a homogeneous mixture was observed, a spatula of DMAP was added. The reaction mixture was stirred at rt for 18 h and complete conversion was confirmed via TLC (CHCl_3_/MeOH/H_2_O 14:7:1, LP/Et_2_O 1:2). Excessive reagent was quenched by the addition of MeOH (8 cm^3^) under ice-bath cooling. After dilution with DCM (200 cm^3^), extraction with 1 N HCl was performed until the aq. phase remained acidic. The organic layer was further washed with sat. NaHCO_3_ (150 cm^3^) and brine. After drying over MgSO_4_, the solution was evaporated to dryness. To obtain the *gluco*-isomer **17** in pure form, repeated flash column chromatography (SiO_2_, gradient of Et_2_O in LP from 60 to 75%) was performed. This afforded the d-*gluco*-nonenitol peracetate **17** as a colorless solid (1.08 g, 8% yield over all steps starting from d-mannose (**5**)). Analytical data for **17** (peracetate): m.p.: 89.0–90.0 °C (LP/Et_2_O); *R*_f_ = 0.47 (LP/Et_2_O 1:2); [*α*]_D_^20^ =  + 4.5° cm^2^ g^−1^ (*c* = 1.0, CHCl_3_); ^1^H NMR (400 MHz, CDCl_3_): *δ* = 5.83 (ddd, *J* = 16.8, 10.6, 6.0 Hz, 1H, H-2), 5.41–5.22 (m, 6H, 6 × CH), 5.11 (dd, *J* = 7.6, 2.6 Hz, 1H, CH), 5.01 (ddd, *J* = 8.4, 5.4, 2.9 Hz, 1H, H-8), 4.20 (dd, *J* = 12.5, 2.9 Hz, 1H, H-9a), 4.03 (dd, *J* = 12.5, 5.4 Hz, 1H, H-9b), 2.10 (s, 3H, C(=O)C*H*_3_), 2.09 (s, 3H, C(=O)C*H*_3_), 2.07 (s, 6H, 2 × C(=O)C*H*_3_), 2.06 (s, 6H, 2 × C(=O)C*H*_3_), 2.03 (s, 3H, C(=O)C*H*_3_) ppm; ^13^C NMR (101 MHz, CDCl_3_): *δ* = 170.7, 170.1, 169.99, 169.98, 169.8, 169.6 (7 × *C*(=O)CH_3_), 131.5 (C-2), 120.0 (C-1), 72.1, 70.3, 68.4 (C-8), 67.7, 67.4, 67.1 (6 × CH), 62.0 (C-9), 21.00, 20.96, 20.94, 20.86, 20.8, 20.7 (7 × C(=O)*C*H_3_) ppm; HR-MS (^+^ESI-TOF): *m*/*z* calc. for C_23_H_32_NaO_14_ ([M + Na]^+^) 555.1684, found 555.1689.

For structural confirmation, an analytical sample of **17** was deprotected (NaOMe, MeOH) to give compound **16**. Analytical data for **16**: ^1^H NMR (400 MHz, D_2_O): *δ* = 5.90 (ddd, *J* = 17.6, 10.4, 7.4 Hz, 1H, H-2), 5.41 (dt, *J* = 17.2, 1.2 Hz, 1H, H-1a), 5.33 (d, *J* = 10.5 Hz, 1H, H-1b), 4.26 (t, *J* = 7.7 Hz, 1H, H-3), 3.93 (d, *J* = 9.3 Hz, 1H, CH), 3.88 (dd, *J* = 11.7, 2.4 Hz, 1H, H-9a), 3.82–3.76 (m, 3H, 3 × CH), 3.74 (dd, *J* = 9.5, 1.1 Hz, 1H, CH), 3.71–3.65 (m, 1H, H-9b) ppm; ^13^C NMR (101 MHz, D_2_O): *δ* = 136.2 (C-2), 118.6 (C-1), 74.6 (C-3), 72.4, 70.9, 69.0, 68.3 (5 × CH), 63.3 (C-9) ppm.

### d*-Erythro-*l*-gluco*-octitol (**15**, C_8_H_18_O_8_)

*Step 1—Ozonolysis and reduction*: The *gluco*-nonenitol peracetate **17** (1.02 g, 2.06 mmol, 1.00 eq.) was dissolved in DCM/MeOH (3:1, dry, 100 cm^3^) and the solution chilled to −78 °C using a liquid N_2_/acetone bath. Then, ozone was bubbled through until the solution turned dark blue. The reaction mixture was stirred for 30 min at −78 °C, then oxygen was bubbled through until the blue color diminished. TLC analysis (LP/Et_2_O 1:7) was performed showing complete conversion of the starting material. NaBH_4_ (229 mg, 6.05 mmol, 3.00 eq.) was added and stirring was continued at rt to reduce the formed ozonides. After 30 min, TLC analysis and LC–MS confirmed the absence of any intermediary formed aldehyde. Therefore, AcOH (14 cm^3^) was added and the reaction mixture was evaporated in vacuo giving a colorless crude residue (2.3 g).

*Step 2—Acetylation*: The residue (2.3 g) was taken up pyridine (9.2 cm^3^) and Ac_2_O (1.14 cm^3^, 12.1 mmol, 6.00 eq.) was added under ice-bath cooling. After a homogeneous solution was formed, a spatula tip of DMAP was added and the reaction mixture was stirred at rt for 18 h. Complete conversion to the peracetate was confirmed via LC–MS. Excessive reagent was quenched by the addition of MeOH (0.5 cm^3^) under ice-bath cooling. The reaction mixture was diluted with DCM (150 cm^3^) and extracted with 1 N HCl until the aq. phase remained acidic. The combined aq. phases were extracted with DCM once (10 cm^3^) and the pooled organic phases were washed with sat. aq. NaHCO_3_ and brine. After drying over MgSO_4_ the solvent was evaporated giving the *gluco*-octitol peracetate (1.07 g) that was used in the next step without further purification.

*Step 3—Deacetylation*: The peracetate (1.07 g) was taken up in MeOH (11 cm^3^) and NaOMe (30% in MeOH, 25 mm^3^, pH ~ 9) was added dropwise at rt under stirring. After 30 min, reaction monitoring via LC–MS indicated complete conversion to the desired sugar alcohol. The reaction mixture was neutralized by the addition of Dowex-H^+^ and water was added to dissolve the formed octitol. The resin was filtered off and the filter cake washed with additional water. The filtrate was lyophilized to obtain the *gluco*-octitol **15** as a colorless solid (353 mg, 72% yield over 3 steps), pure according to NMR. M.p.: 171.8 °C (H_2_O, from DSC); [*α*]_D_^20^ =  + 2.4° cm^2^ g^−1^ (*c* = 1.0, H_2_O); ^1^H NMR (400 MHz, D_2_O): *δ* = 3.95 (d, *J* = 9.4 Hz, 1H), 3.92–3.85 (m, 3H), 3.83–3.74 (m, 4H), 3.71–3.61 (m, 2H) ppm; ^13^C NMR (101 MHz, D_2_O): *δ* = 73.2, 70.9, 69.9, 69.7, 69.0, 68.4, 63.3 (C-1/C-8), 62.4 (C-1/C-8) ppm; HR-MS (^+^ESI-TOF): *m*/*z* calc. for C_8_H_18_NaO_8_ ([M + Na]^+^) 265.0894, found 265.0900.

*Purification for DSC*: The product was recrystallized from H_2_O with slow cooling rate to allow a better growth of crystals that can be further analyzed in STA measurements.

## Supplementary Information

Below is the link to the electronic supplementary material.

Supplementary file1 (PDF 2493 KB)

## Data Availability

The authors confirm that all data underlying this article is available within the main manuscript and its supporting information.
