# Supplementary material for: The synthesis of higher-carbon sugar alcohols via indium-mediated acyloxyallylation as potential phase change materials
Source: Monatsh Chem. 2023 Dec 16;156(1):51–63. doi: 10.1007/s00706-023-03136-6 (PMC11703932; doi:10.1007/s00706-023-03136-6)
Supplement: Supplementary file 1 — Supplementary file1 (PDF 2493 KB) [file 706_2023_3136_MOESM1_ESM.pdf]

Supporting Information

The synthesis of higher-carbon sugar alcohols *via* indium-mediated acyloxyallylation as potential phase change materials

Markus Draskovits[1], Nina Biedermann[1], Marko D. Mihovilovic[1], Michael Schnürch*[,1], Christian Stanetty*[,1]

Institute of Applied Synthetic Chemistry, TU Wien, Getreidemarkt 9/163, 1060 Vienna, Austria

E-Mail: michael.schnuerch@tuwien.ac.at, christian.stanetty@tuwien.ac.at

Table of content

General information.......... 2

Structural elucidation of nonenitols from elongation of D-mannose.......... 3

Spectra.......... 4

STA measurements.......... 15

References.......... 17

General information

^{1}H-NMR and ^{13}C-NMR spectra were recorded at ambient temperature (25 °C) in the solvent indicated using a Bruker Avance Ultra Shield 400 MHz and an Avance III HD 600 MHz spectrometer. Processing of the data was performed with standard software and all spectra were calibrated to the solvent residual peak. Chemical shifts (δ) are reported in ppm, coupling constants (*J*) in hertz (Hz) and multiplicities are assigned as s = singlet, d = doublet, t = triplet, q = quartet, m = multiplet, bs = broad singlet etc. All assignments are based on 2D-sepctra (COSY, phase sensitive HSQC, HMBC – depending on the molecule).
Simultaneous thermal analysis (STA) combining differential scanning calorimetry (DSC, blue curve) and thermogravimetric analysis (TG, green curve) measurements were performed on a Netzsch STA 449 F1 Jupiter under nitrogen atmosphere with a heating and cooling rate of 10 K/min if not stated otherwise. Samples were measured using Al pans with a hole in the lid. Latent heats of fusion were determined by linear integration of the peak appearing in the DSC curve, melting points from the extrapolated onset.

Compounds were named according to IUPAC systematic standards, in general. When it comes to higher-carbon sugar species (more than six carbon atoms), names were generated by dividing the sugar species into groups of up to four chiral centers consequently starting from the chiral center next to the former reducing end (on the right for all displayed structures). To these groups, configurational prefixes were assigned, and the name was built up by putting the prefix of the group that is farthest from the right end (C1) first. This group may contain less than four carbon atoms. Numbering of compounds was performed in the same way, always starting with 1 at the former reducing end as shown in the exemplary structures below:

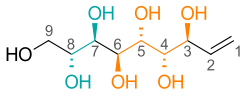

D-*erythro*-L-*manno*-nonenitol

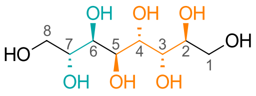

D-*erythro*-L-*manno*-octitol

Spectra are presented in the same order as the substances appear in the experimental part.

Structural elucidation of nonenitols from elongation of D-mannose

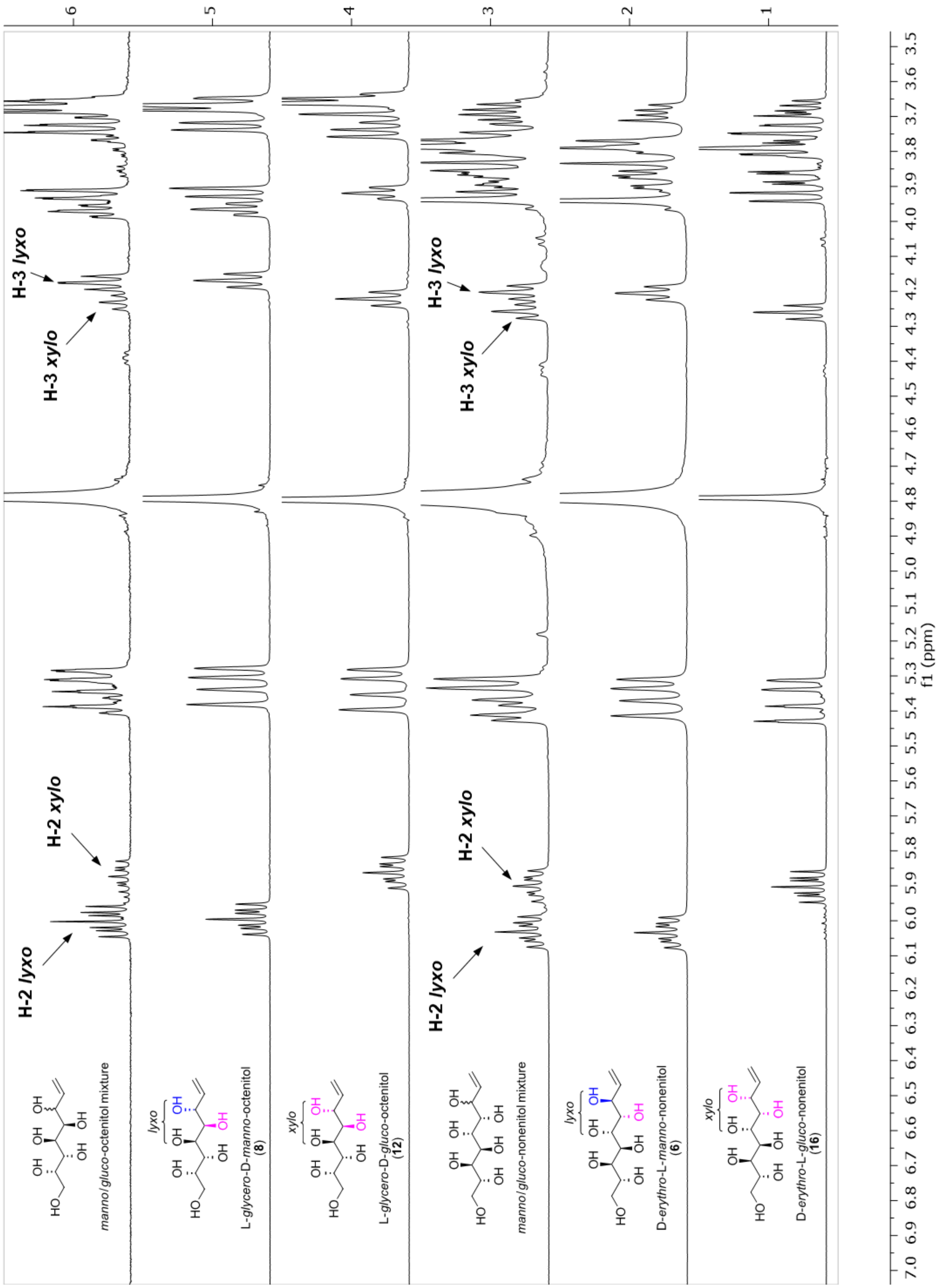

Figure S1. Analysis of nonenitols **6** and **16** by ^{1}H-NMR in comparison to the octenitols **8** and **12**.[1]

Spectra

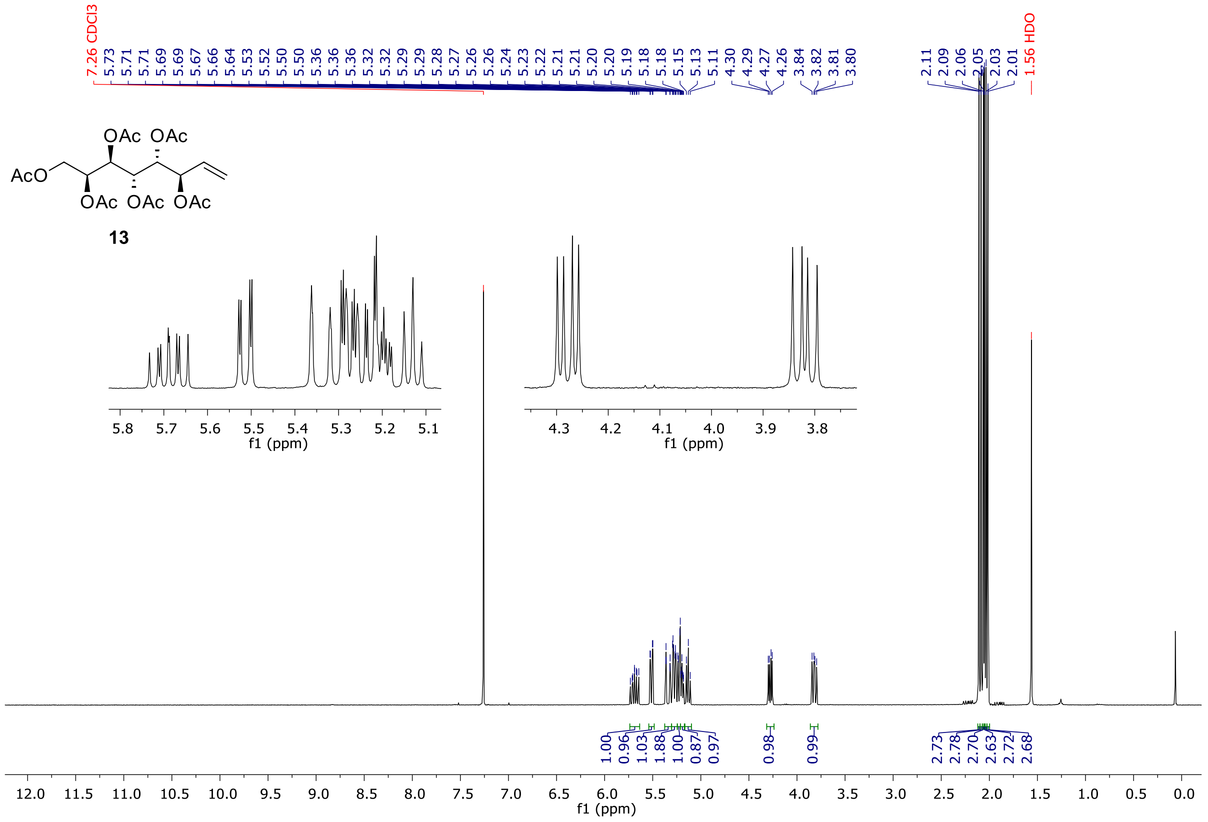

Figure S2. ^{1}H-NMR (400 MHz, $CDCl_3$) of 3,4,5,6,7,8-hexa-*O*-acetyl-1,2-dideoxy-L-*glycero*-D-*manno*-oct-1-enitol (**13**)

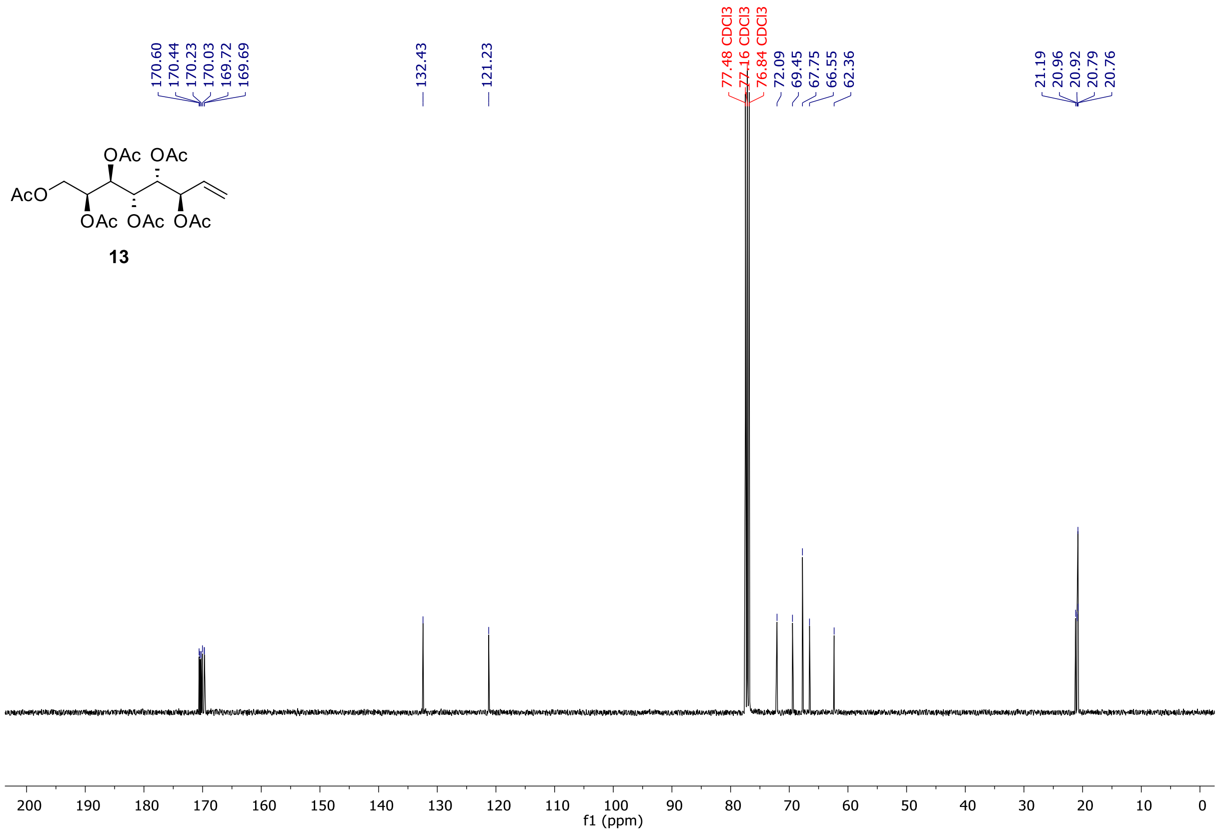

Figure S3. ^{13}H-NMR (400 MHz, $CDCl_3$) of 3,4,5,6,7,8-hexa-*O*-acetyl-1,2-dideoxy-L-*glycero*-D-*manno*-oct-1-enitol (**13**)

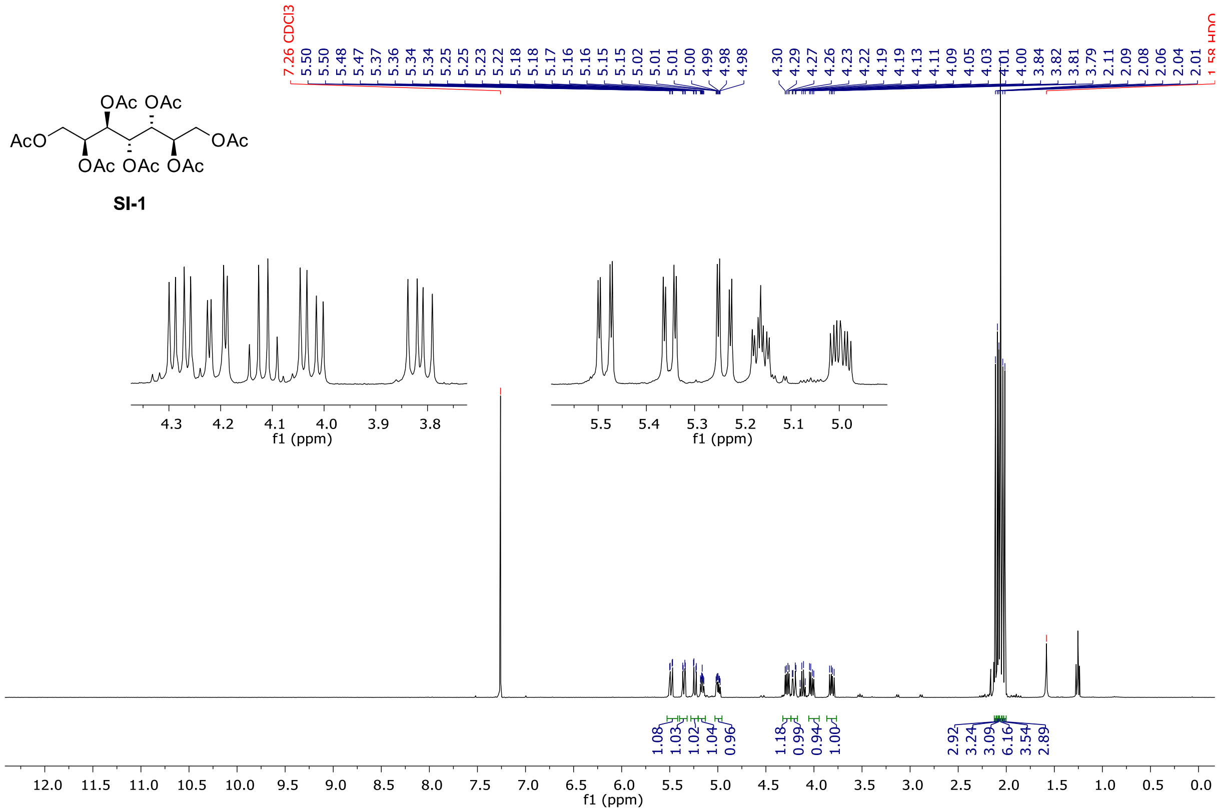

Figure S4. ^{1}H-NMR (400 MHz, $CDCl_3$) of 1,2,3,4,5,6,7-hepta-*O*-acetyl-L-*glycero*-D-*manno*-heptitol (**SI-1**)

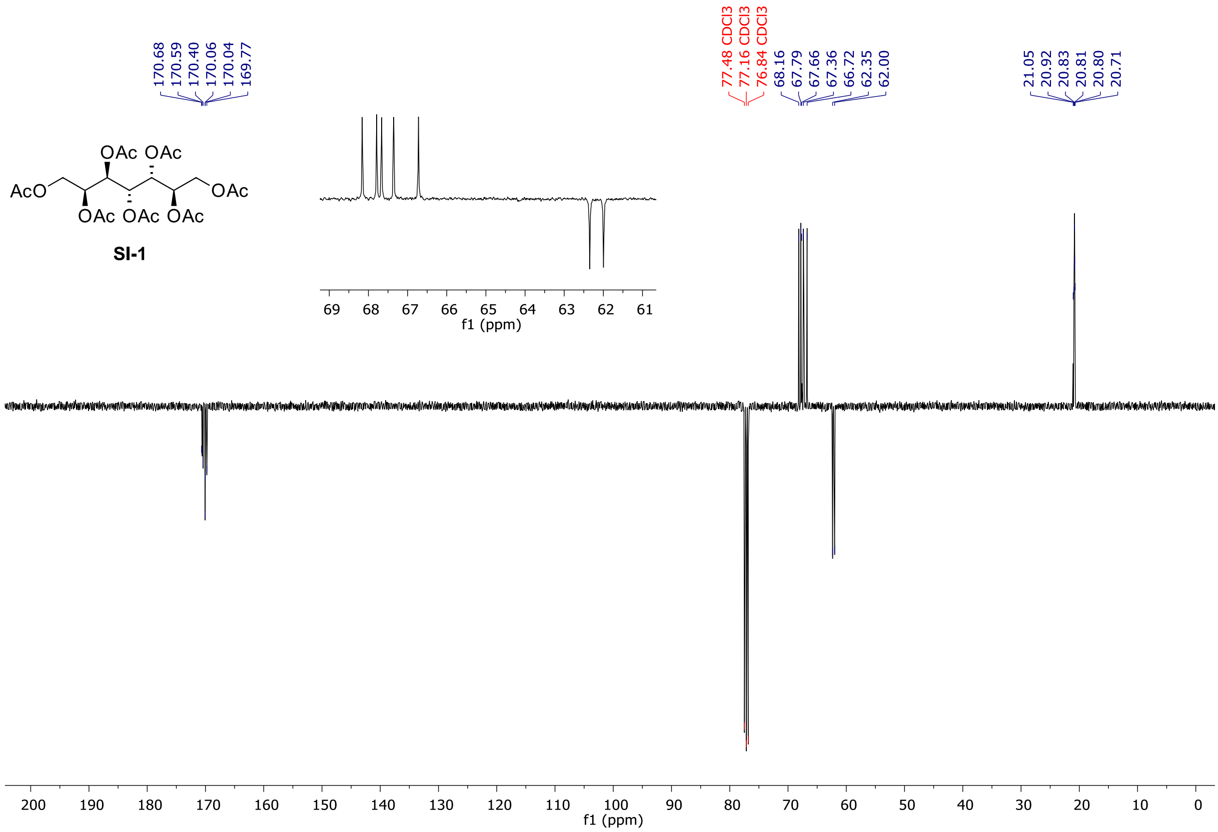

Figure S5. ^{13}C-NMR (101 MHz, $CDCl_3$) of 1,2,3,4,5,6,7-hepta-*O*-acetyl-L-*glycero*-D-*manno*-heptitol (**SI-1**)

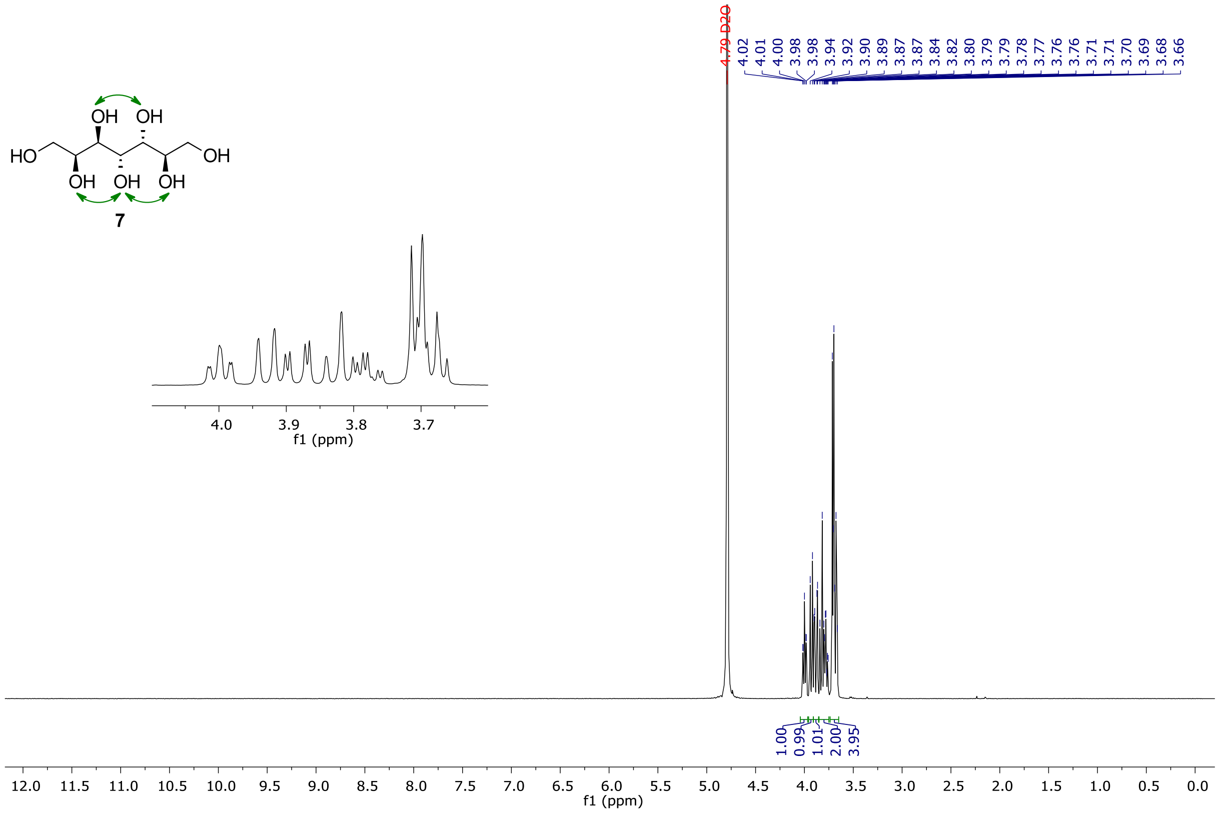

Figure S6. ^{1}H-NMR (400 MHz, D_2O) of L-*glycero*-D-*manno*-heptitol (**7**)

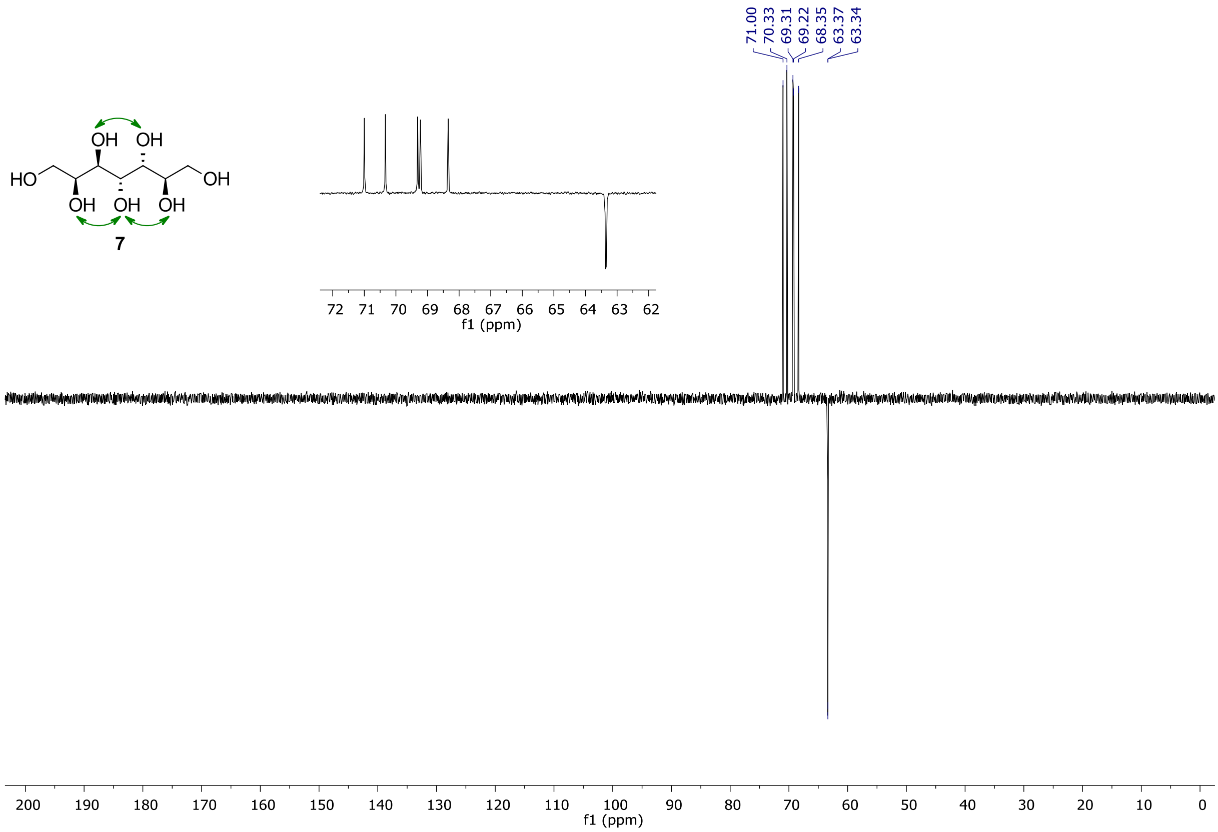

Figure S7. ^{13}C-NMR (101 MHz, D_2O) of L-*glycero*-D-*manno*-heptitol (**7**)

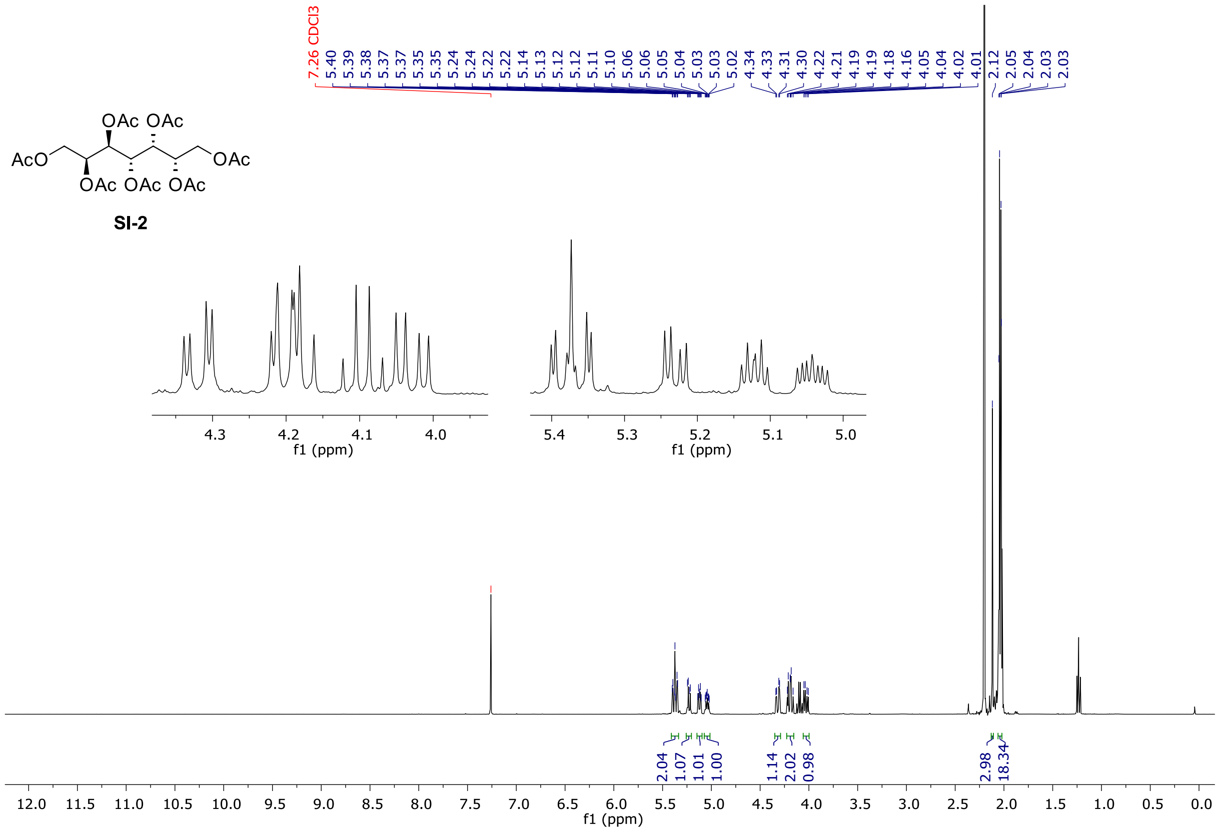

Figure S8. ^{1}H-NMR (400 MHz, $CDCl_3$) of 1,2,3,4,5,6,7-hepta-*O*-acetyl-L-*glycero*-D-*gluco*-heptitol (**SI-2**)

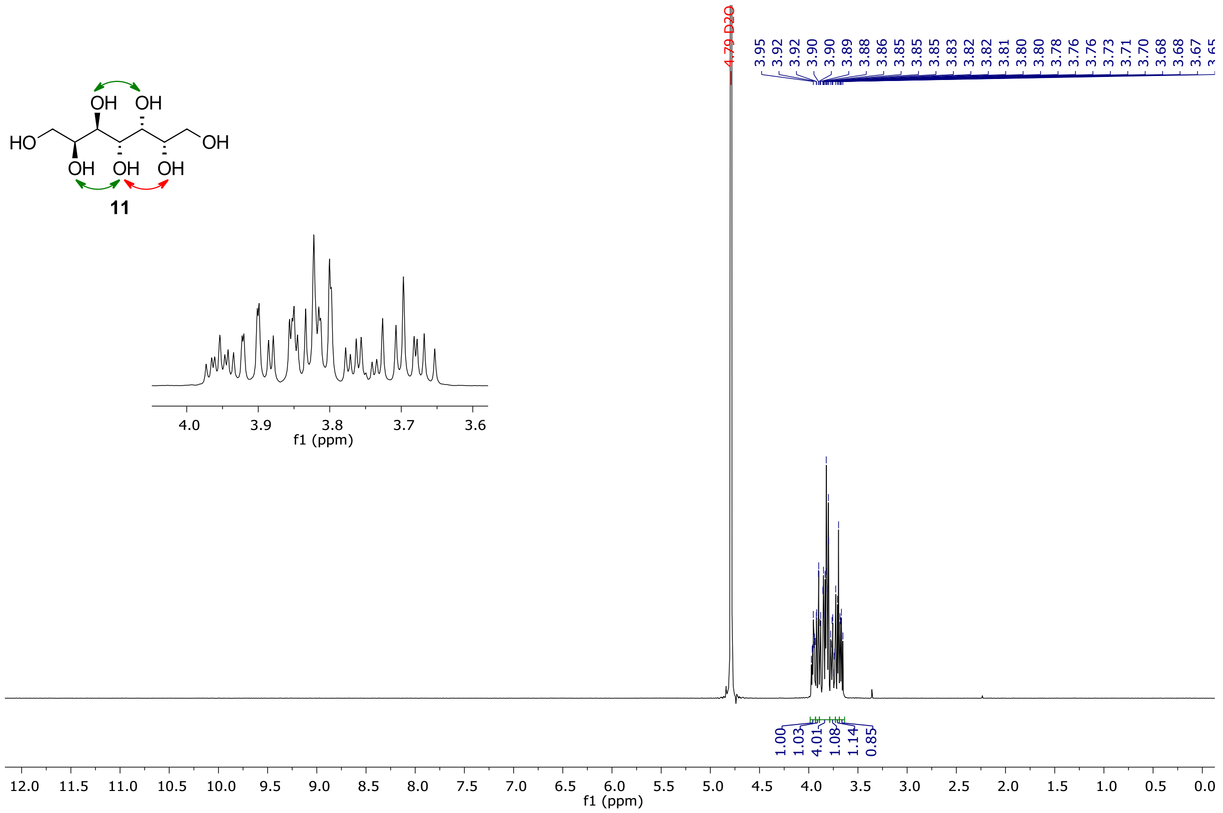

Figure S9. ^{1}H-NMR (400 MHz, D_2O) of L-*glycero*-D-*gluco*-heptitol (**11**)

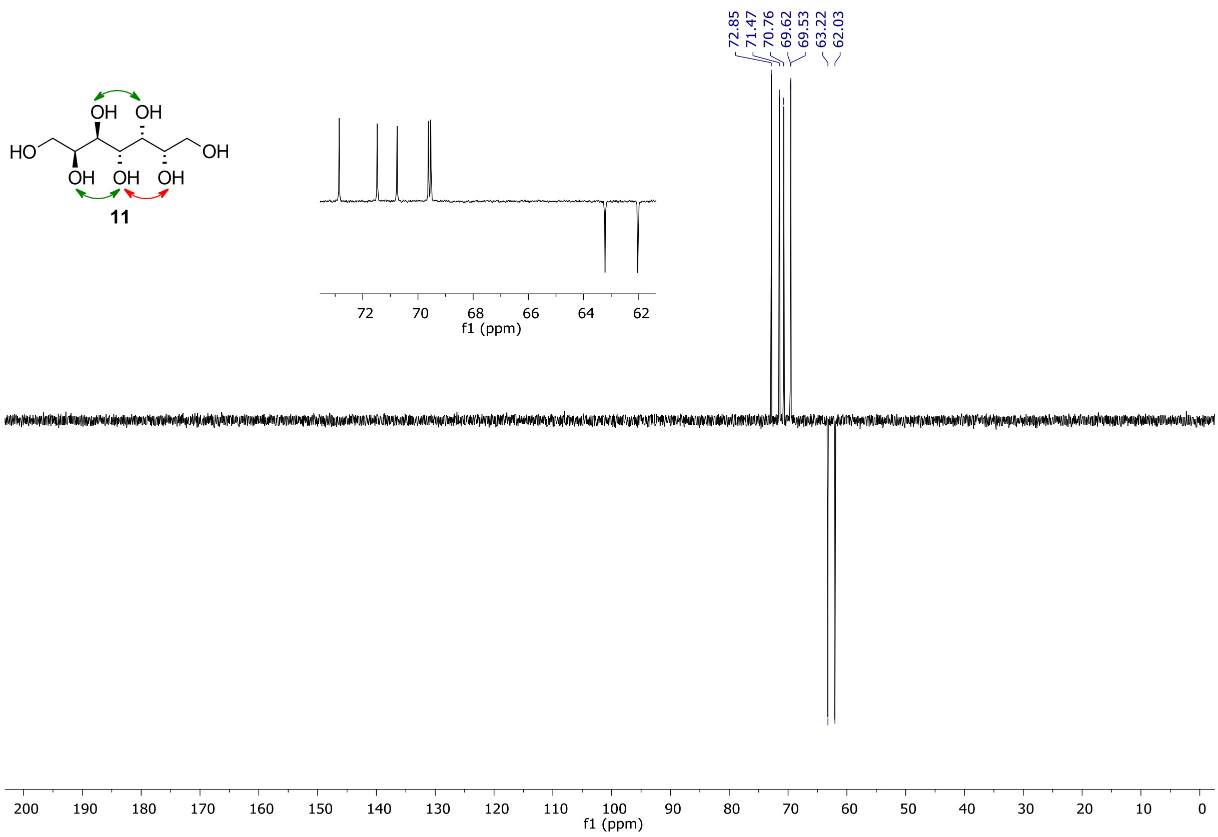

Figure S10. ^{13}C-NMR (101 MHz, D_2O) of L-*glycero*-D-*gluco*-heptitol (**11**)

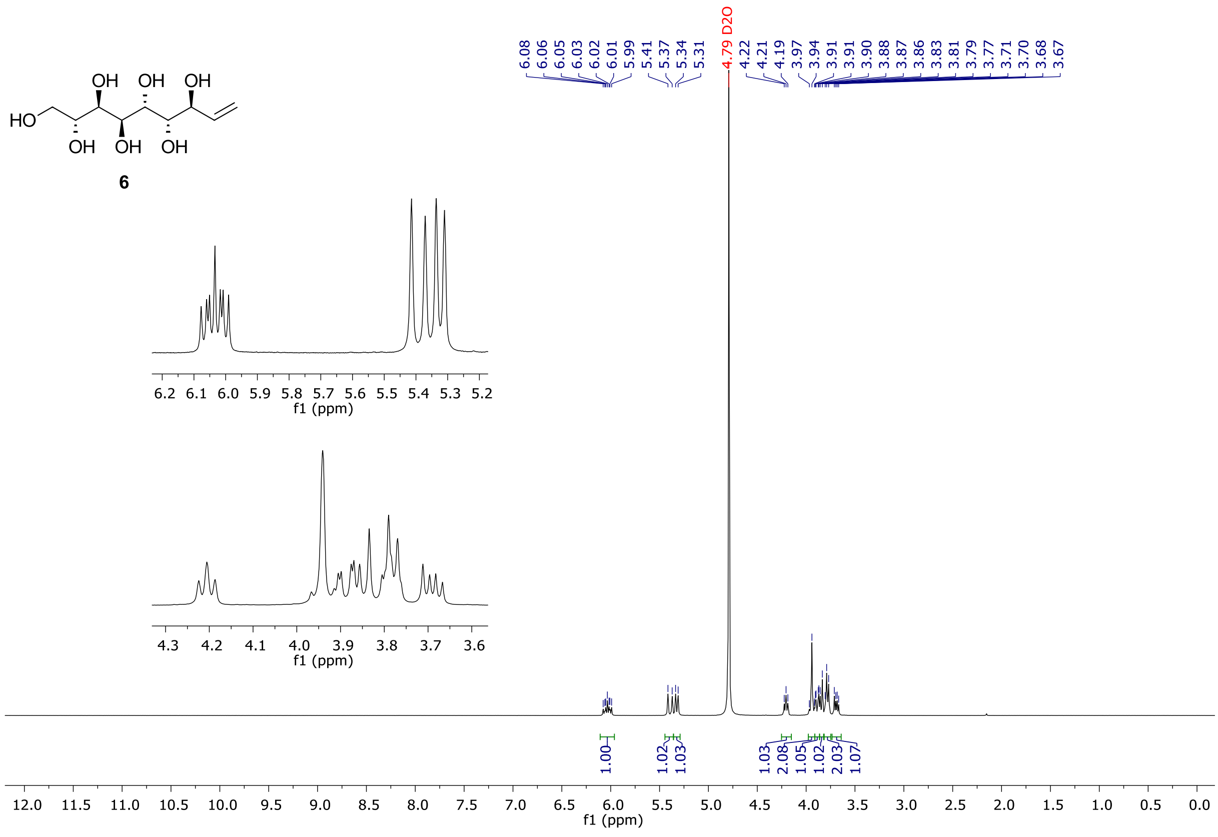

Figure S11. ^{1}H-NMR (400 MHz, D_2O) of 1,2-dideoxy-D-*erythro*-L-*manno*-non-1-enitol (**6**)

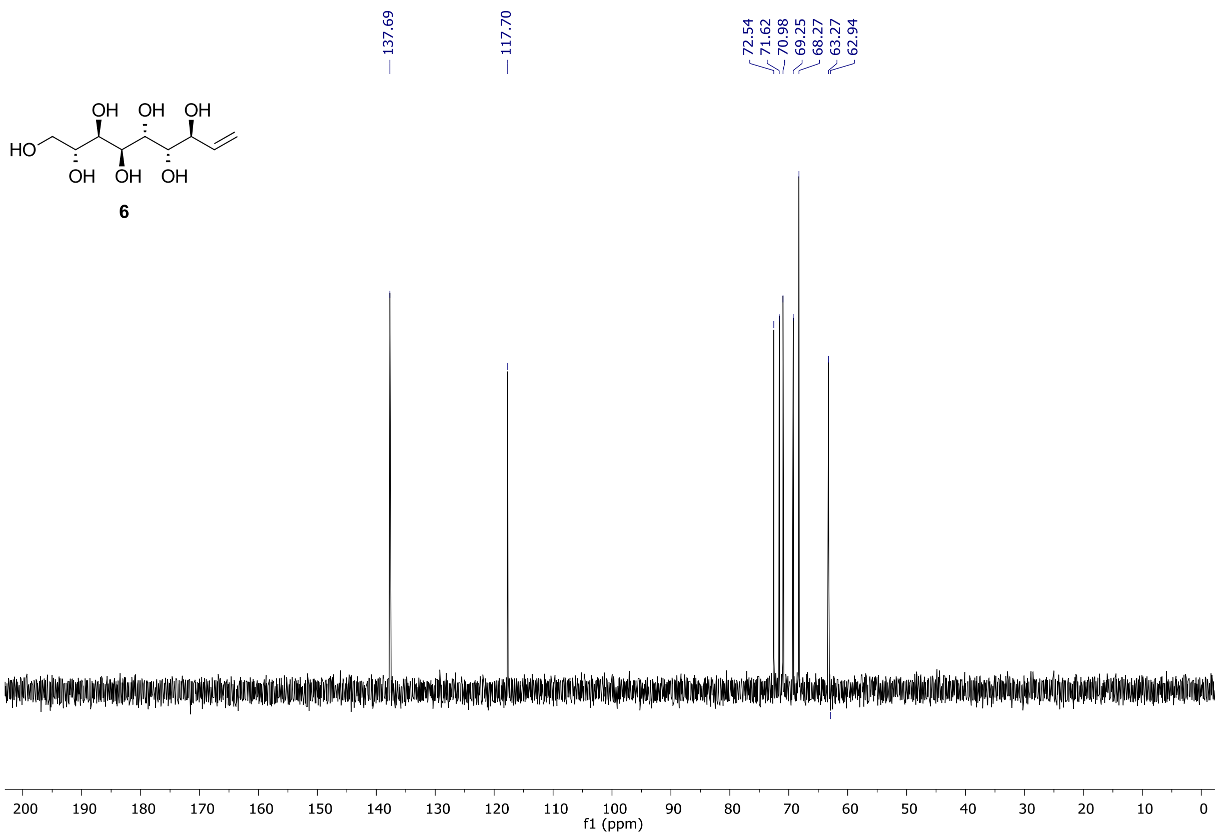

Figure S12. ^{13}C-NMR (101 MHz, D_2O) of 1,2-dideoxy-D-*erythro*-L-*manno*-non-1-enitol (**6**)

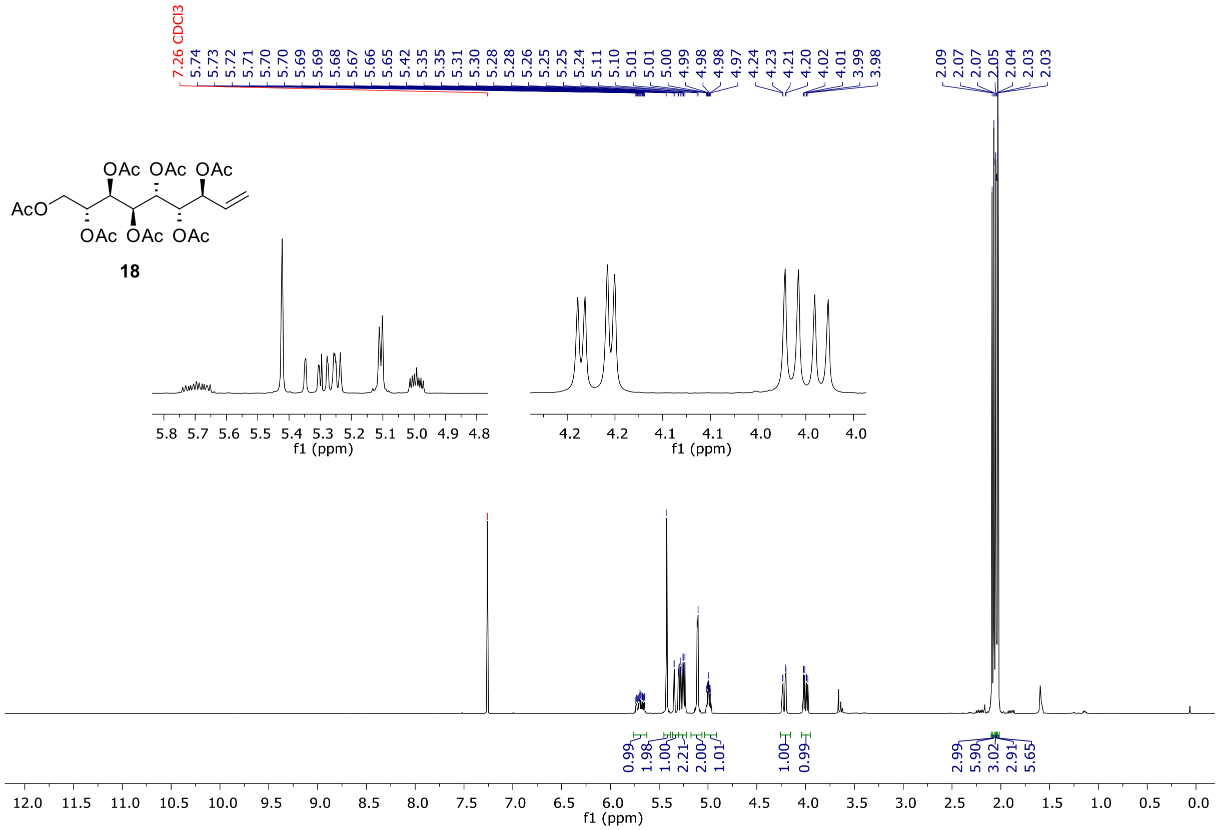

Figure S13. ^{1}H-NMR (400 MHz, $CDCl_3$) of 3,4,5,6,7,8,9-hepta-*O*-acetyl-1,2-dideoxy-D-*erythro*-L-*manno*-non-1-enitol (**18)**

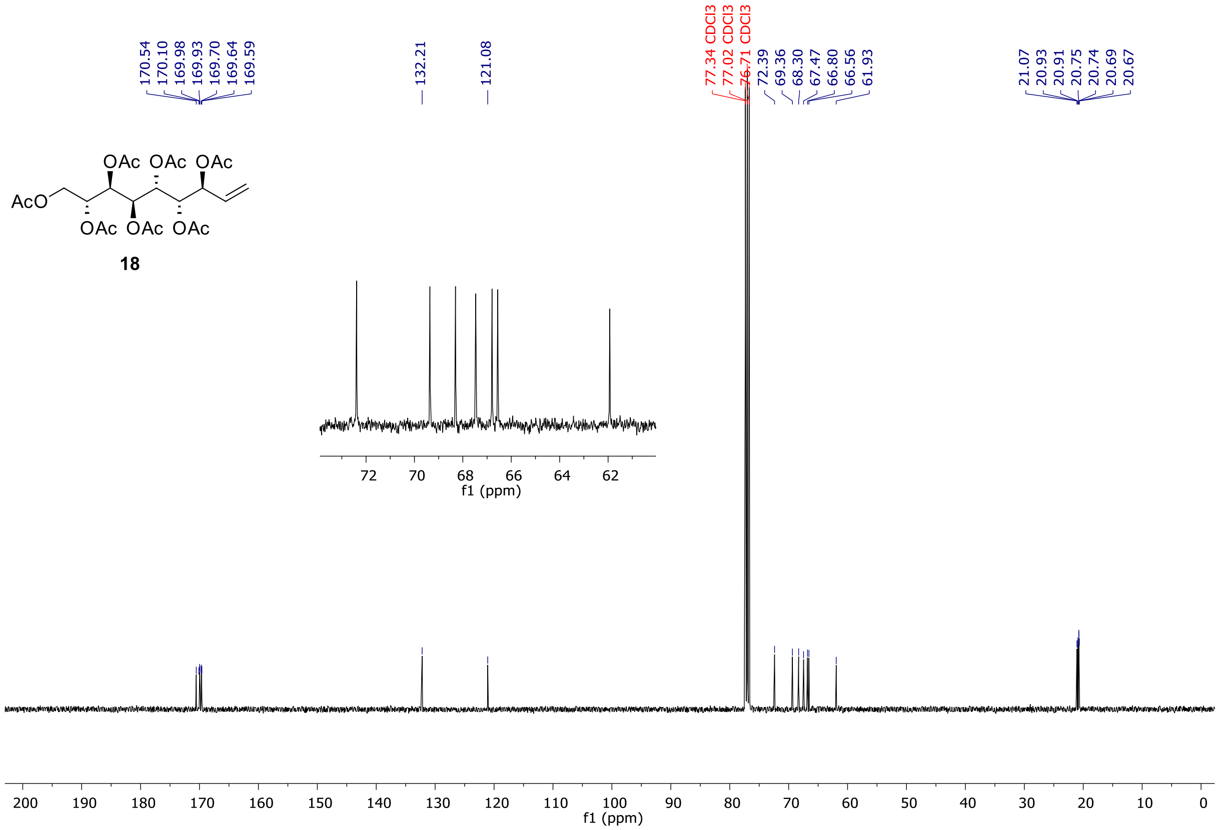

Figure S14. ^{13}C-NMR (101 MHz, $CDCl_3$) of 3,4,5,6,7,8,9-hepta-*O*-acetyl-1,2-dideoxy-D-*erythro*-L-*manno*-non-1-enitol (**18**)

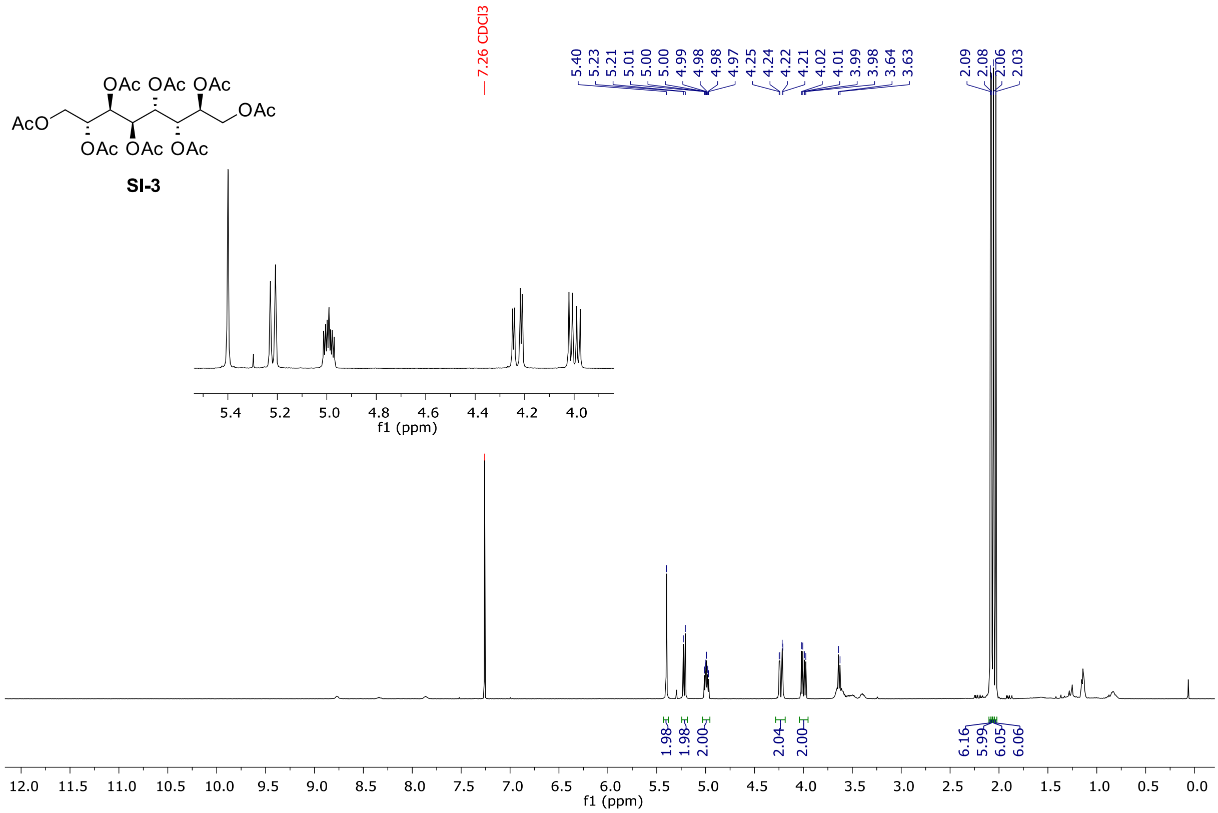

Figure S15. ^{1}H-NMR (400 MHz, $CDCl_3$) of 1,2,3,4,5,6,7,8-octa-*O*-acetyl-D-*erythro*-L-*manno*-octitol (**SI-3**)

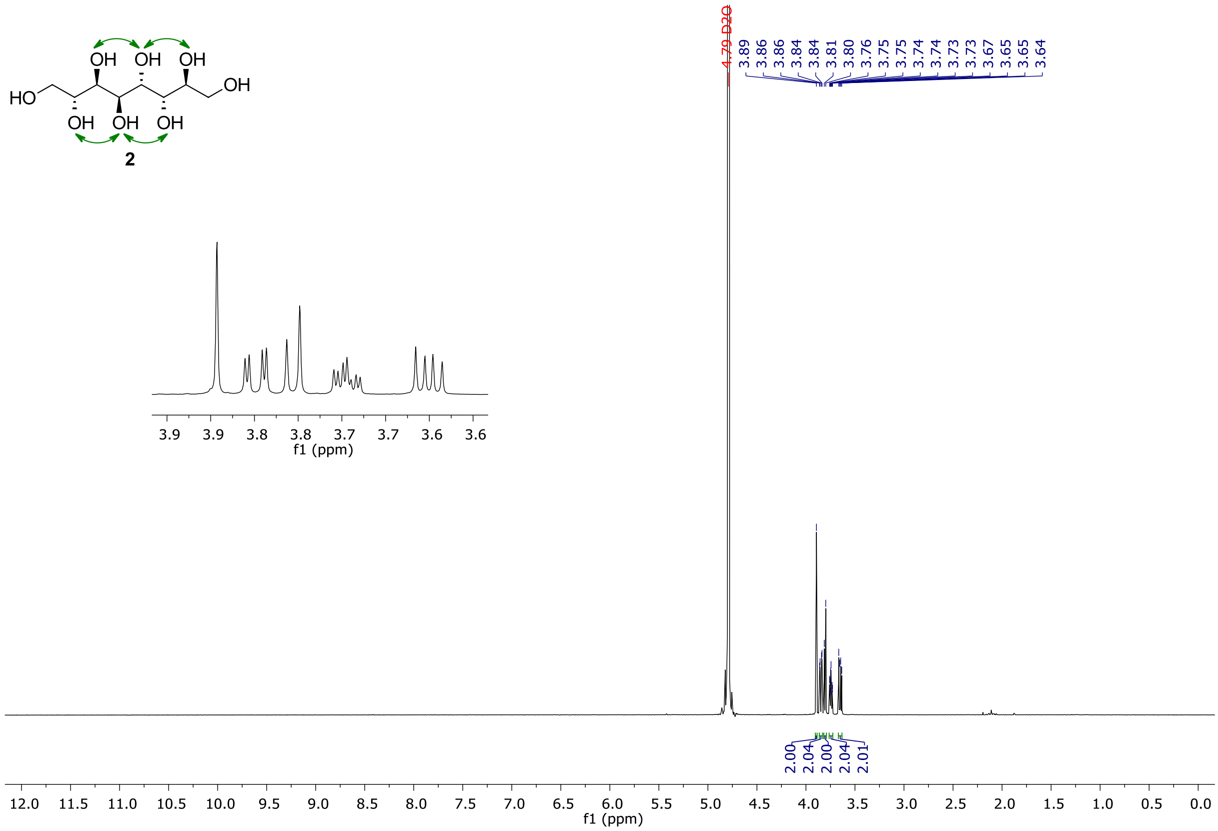

Figure S16. ^{1}H-NMR (600 MHz, D_2O) of *meso*-D-*erythro*-L-*manno*-octitol (**2**)

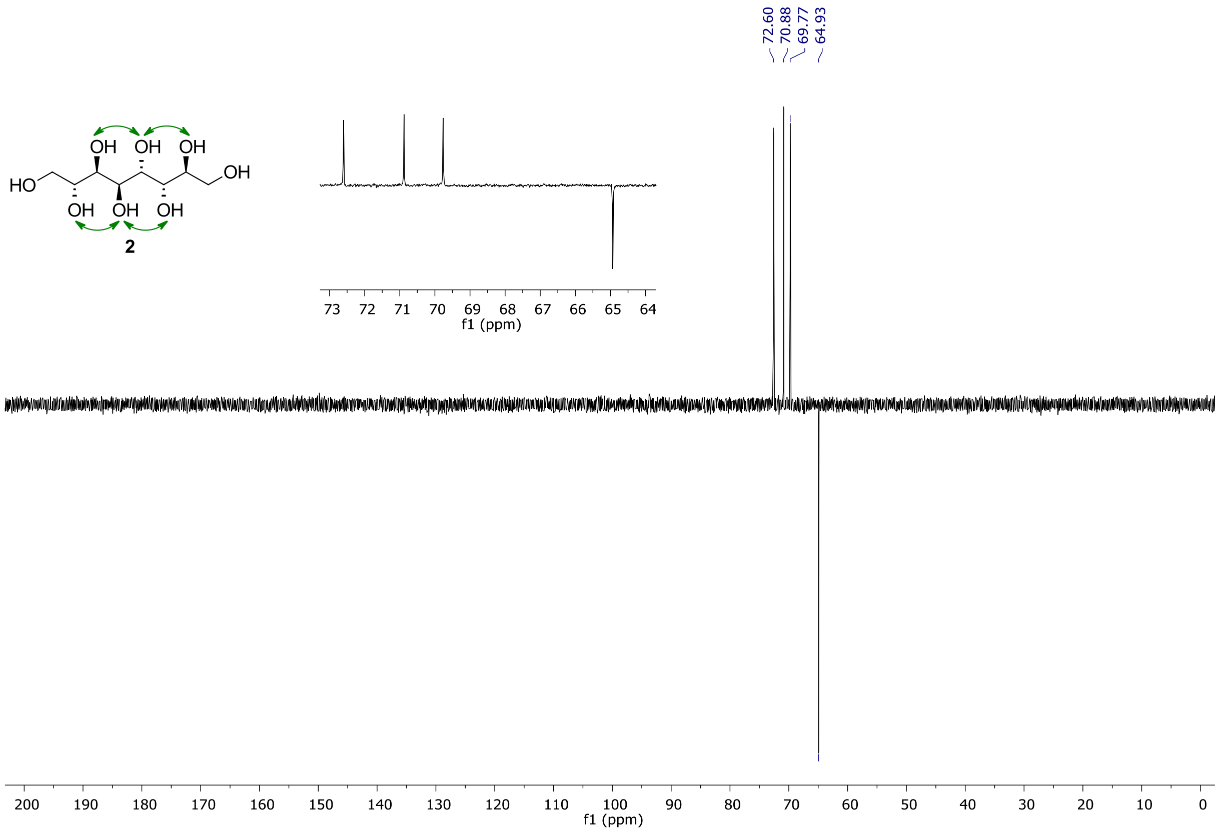

Figure S17. ^{13}C-NMR (151 MHz, D_2O) of *meso*-D-*erythro*-L-*manno*-octitol (**2**)

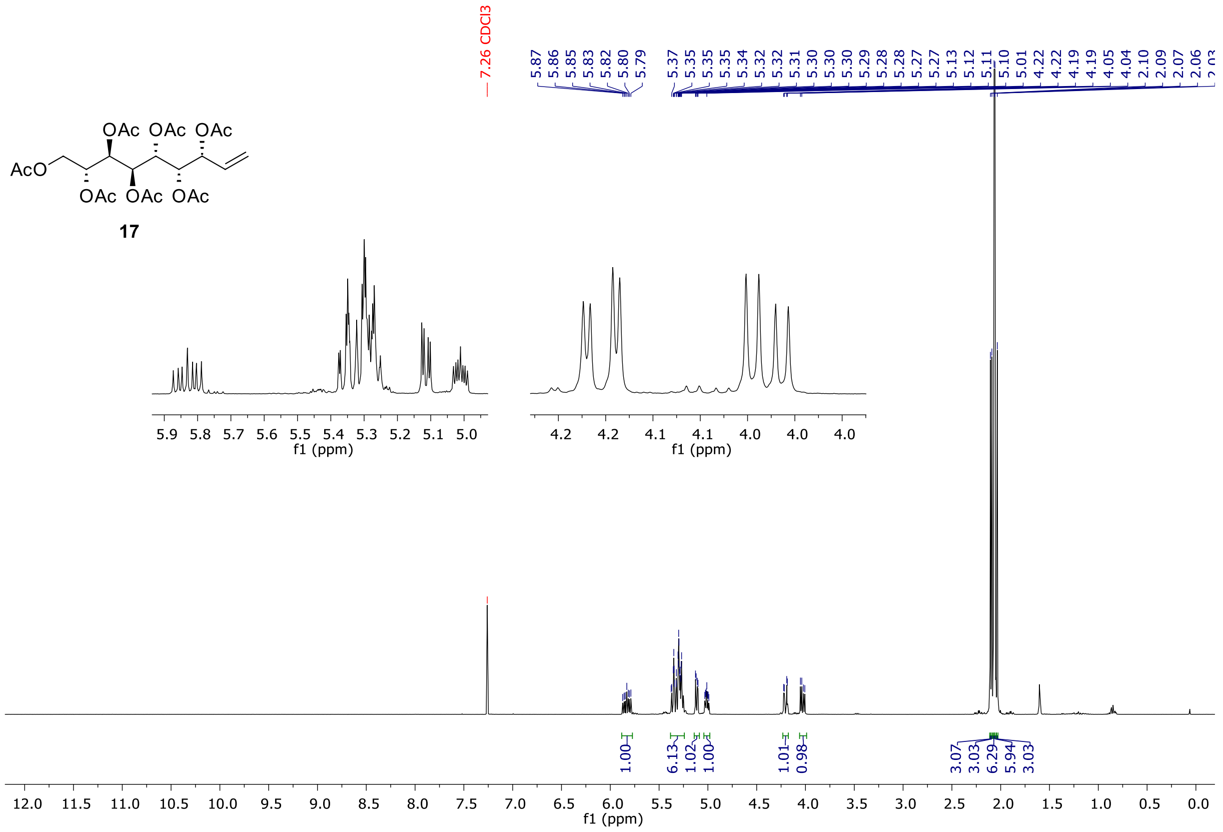

Figure S18. ^{1}H-NMR (400 MHz, $CDCl_3$) of 3,4,5,6,7,8,9-hepta-*O*-acetyl-1,2-dideoxy-D-*erythro*-L-*gluco*-non-1-enitol (**17**)

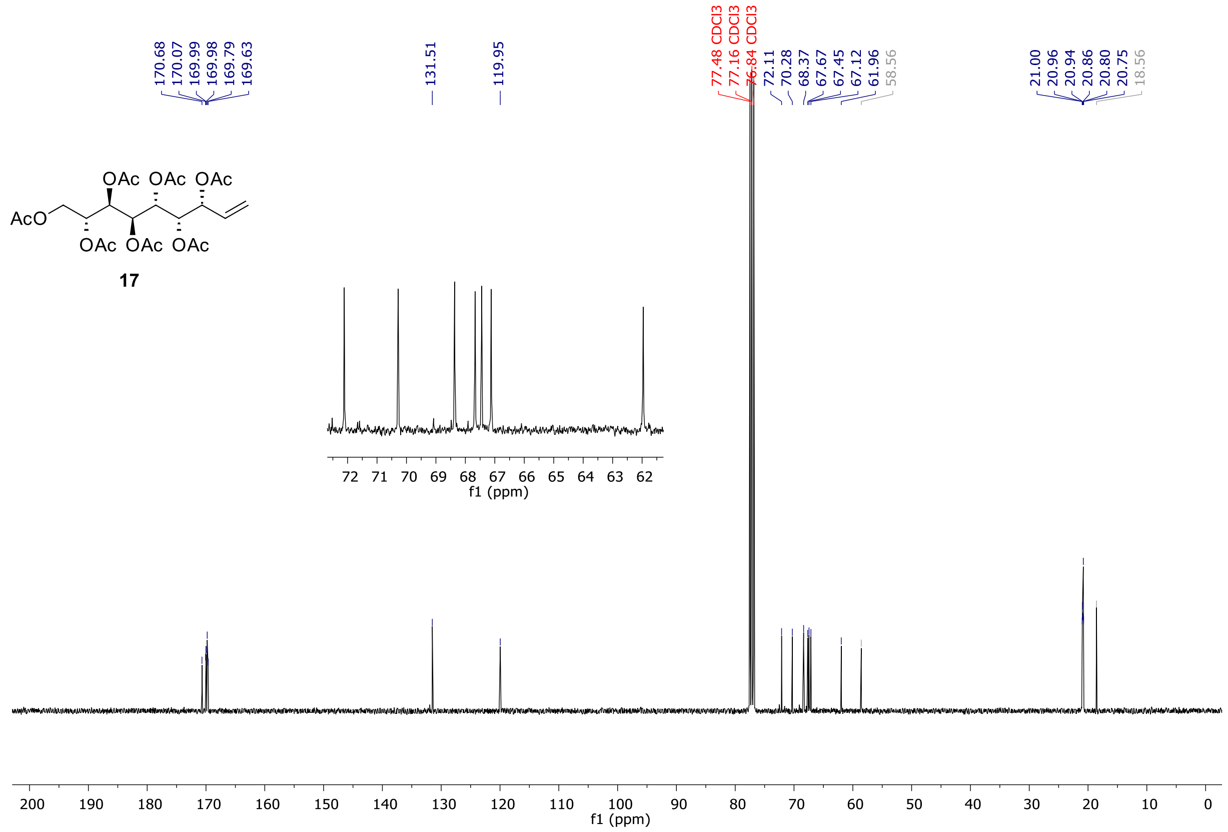

Figure S19. ^{13}C-NMR (101 MHz, $CDCl_3$) of 3,4,5,6,7,8,9-hepta-*O*-acetyl-1,2-dideoxy-D-*erythro*-L-*gluco*-non-1-enitol (**17**)

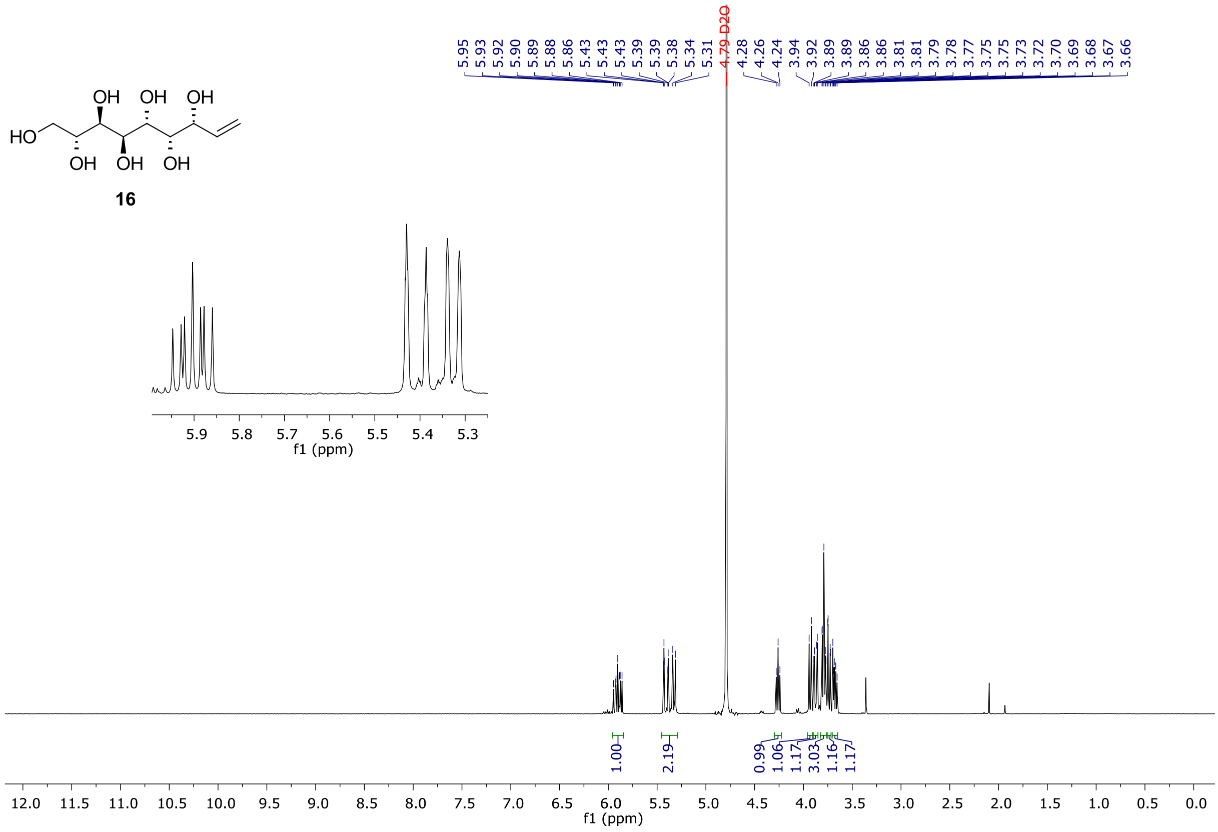

Figure S20. ^{1}H-NMR (400 MHz, D_2O) of 1,2-dideoxy-D-*erythro*-L-*gluco*-non-1-enitol (**16**)

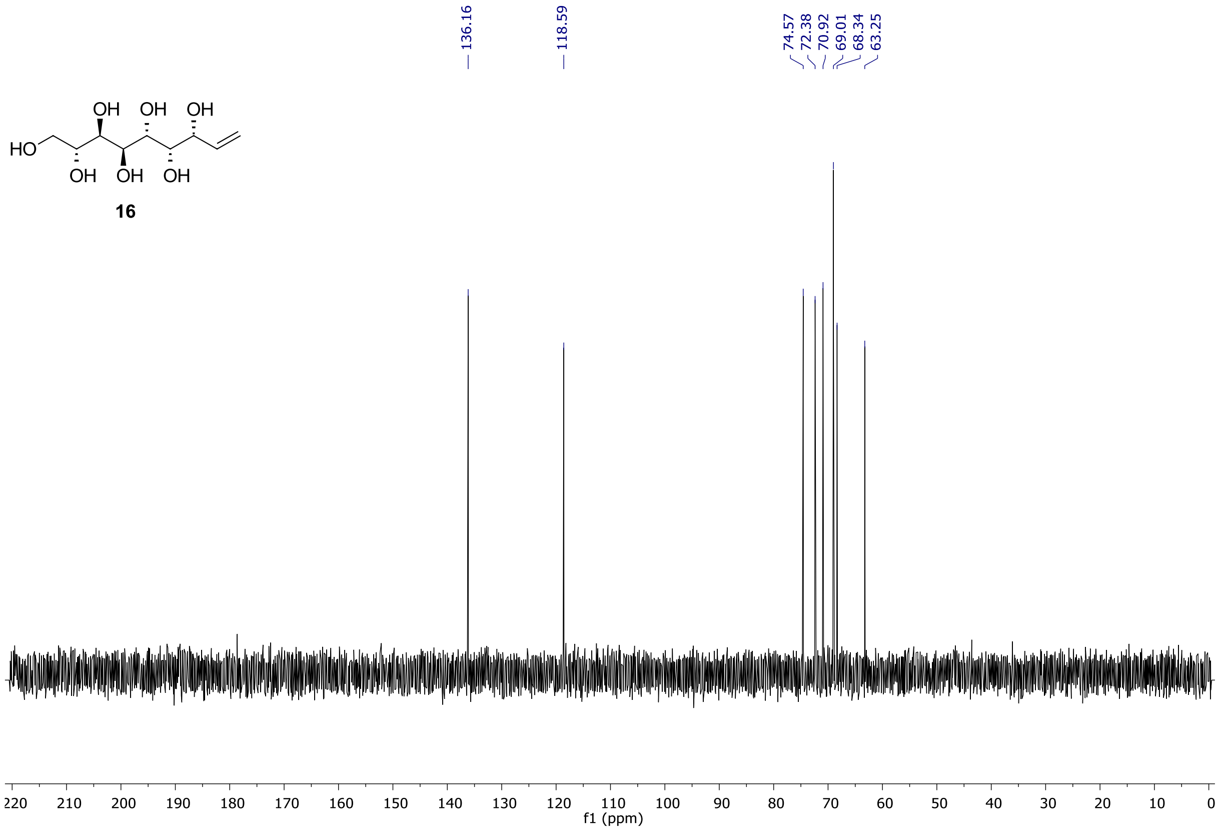

Figure S21. ^{13}C-NMR (101 MHz, D_2O) of 1,2-dideoxy-D-*erythro*-L-*gluco*-non-1-enitol (**16**)

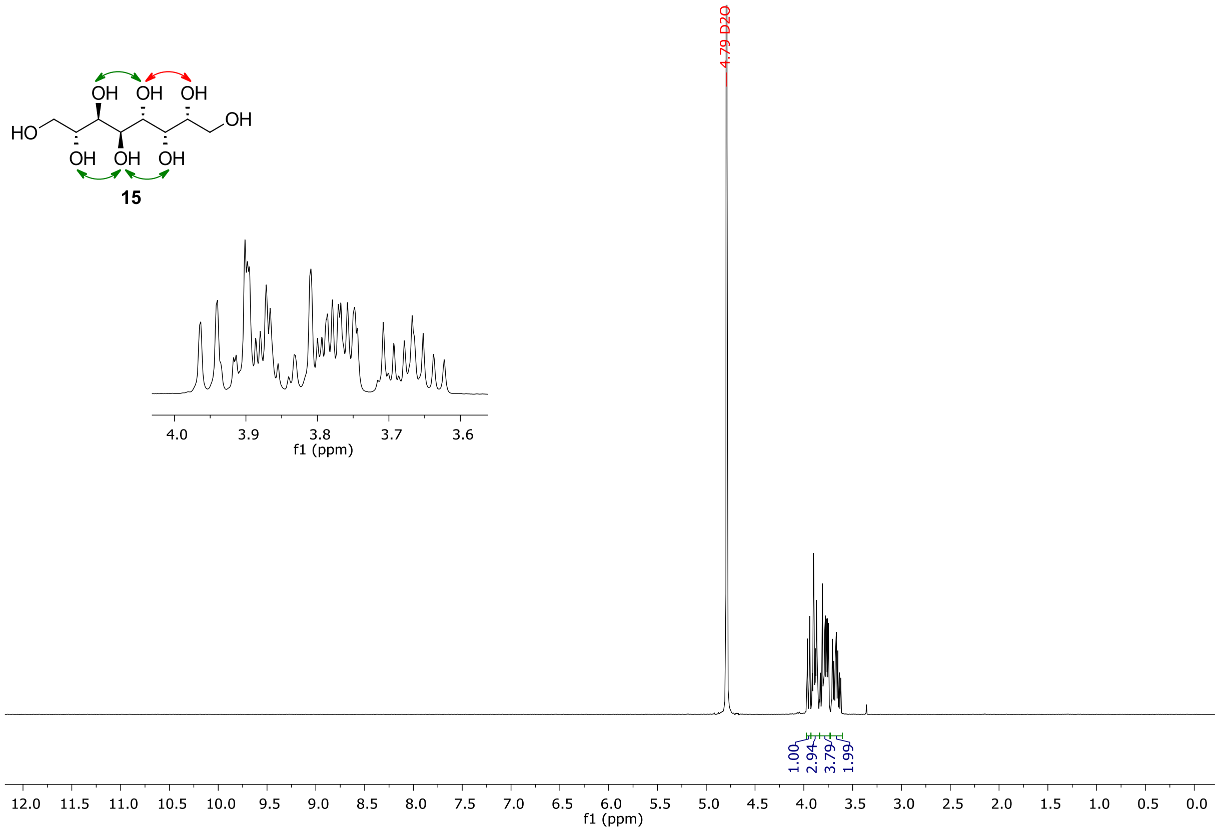

Figure S22. ^{1}H-NMR (400 MHz, D_2O) of D-*erythro*-L-*gluco*-octitol (**15**)

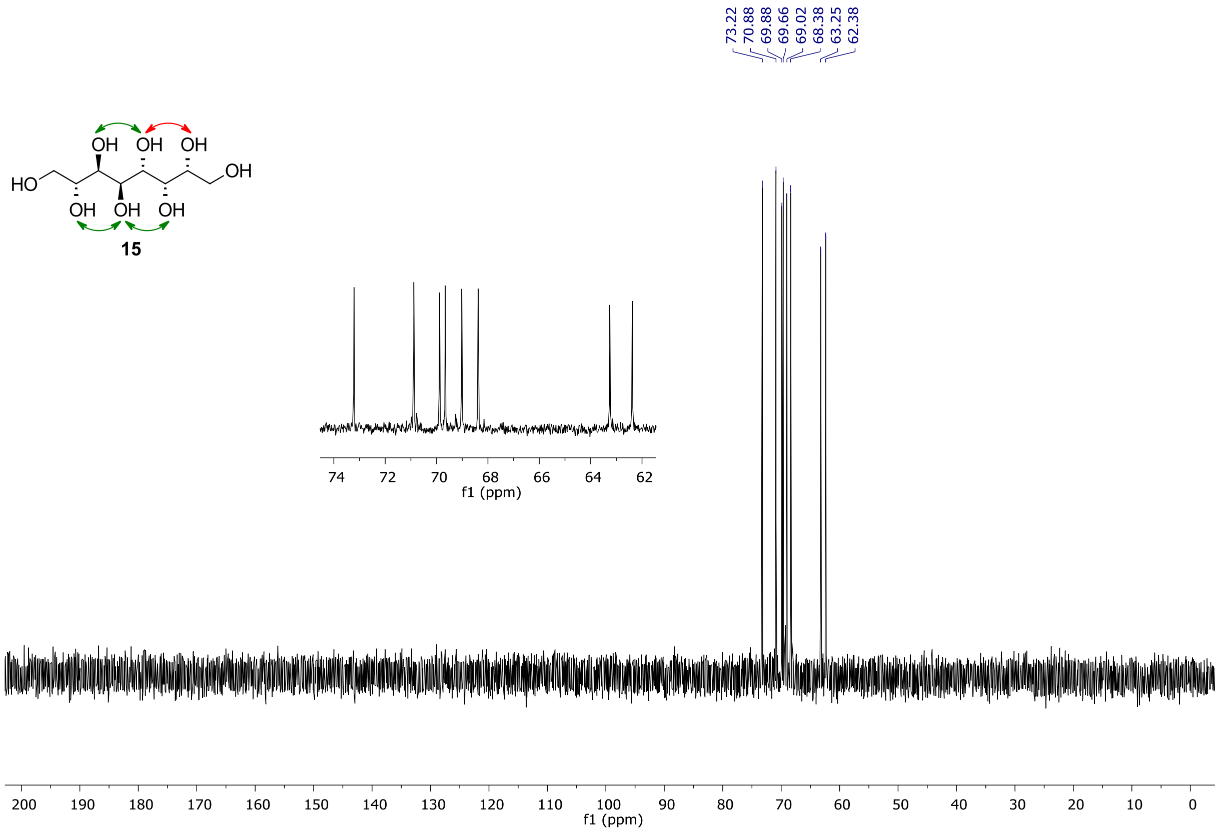

Figure S23. ^{13}C-NMR (101 MHz, D_2O) of D-*erythro*-L-*gluco*-octitol (**15**)

STA measurements

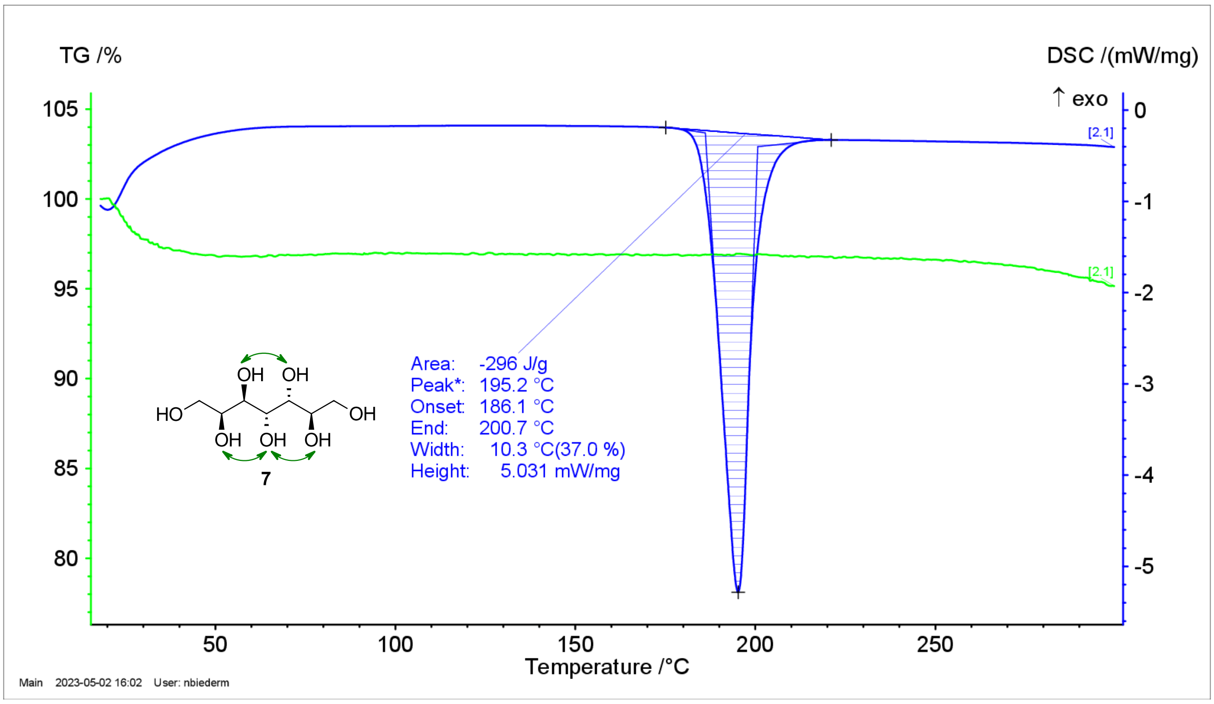

Figure S24. STA measurement of L-*glycero*-D-*manno*-heptitol (**7**)

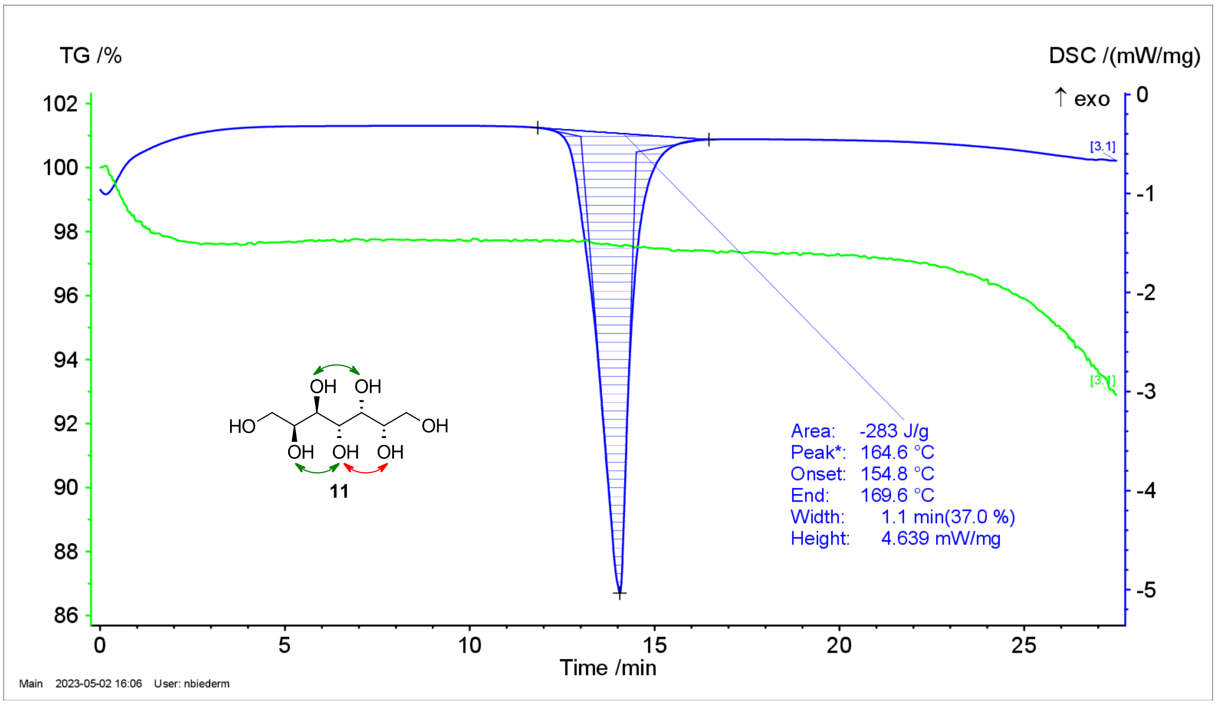

Figure S25. STA measurement of L-*glycero*-D-*gluco*-heptitol (**11**)

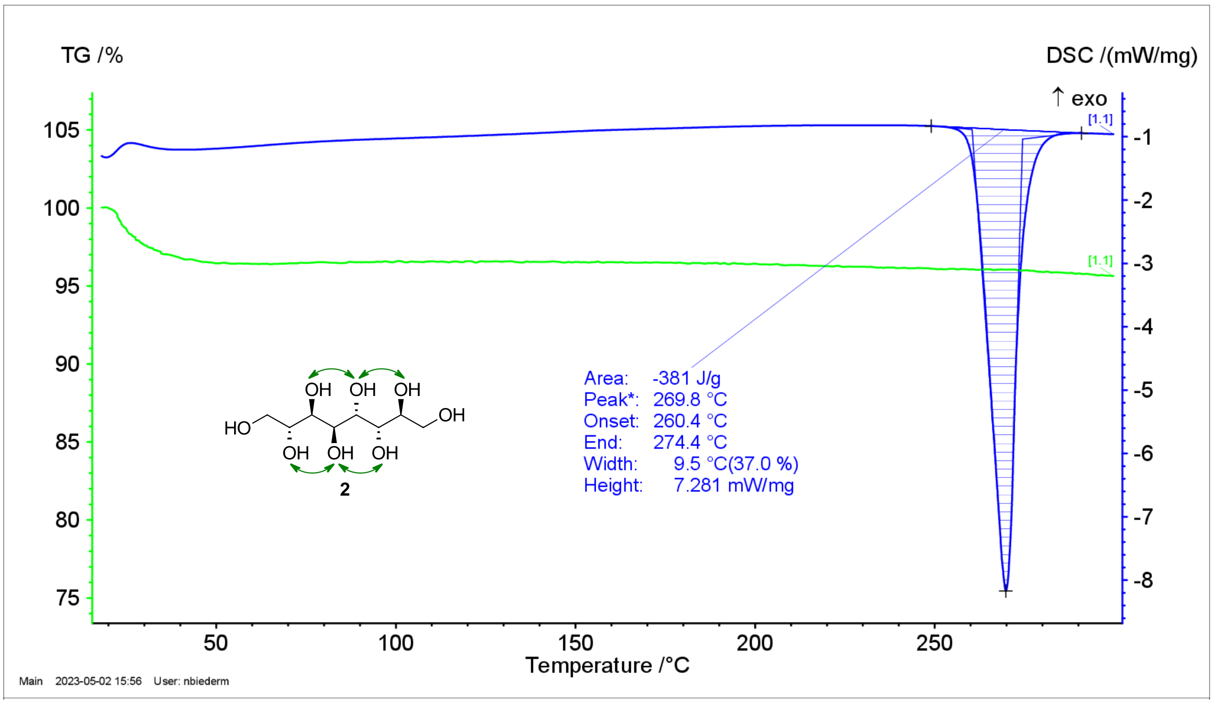

Figure S26. STA measurement of *meso*-D-*erythro*-L-*manno*-octitol (**2**)

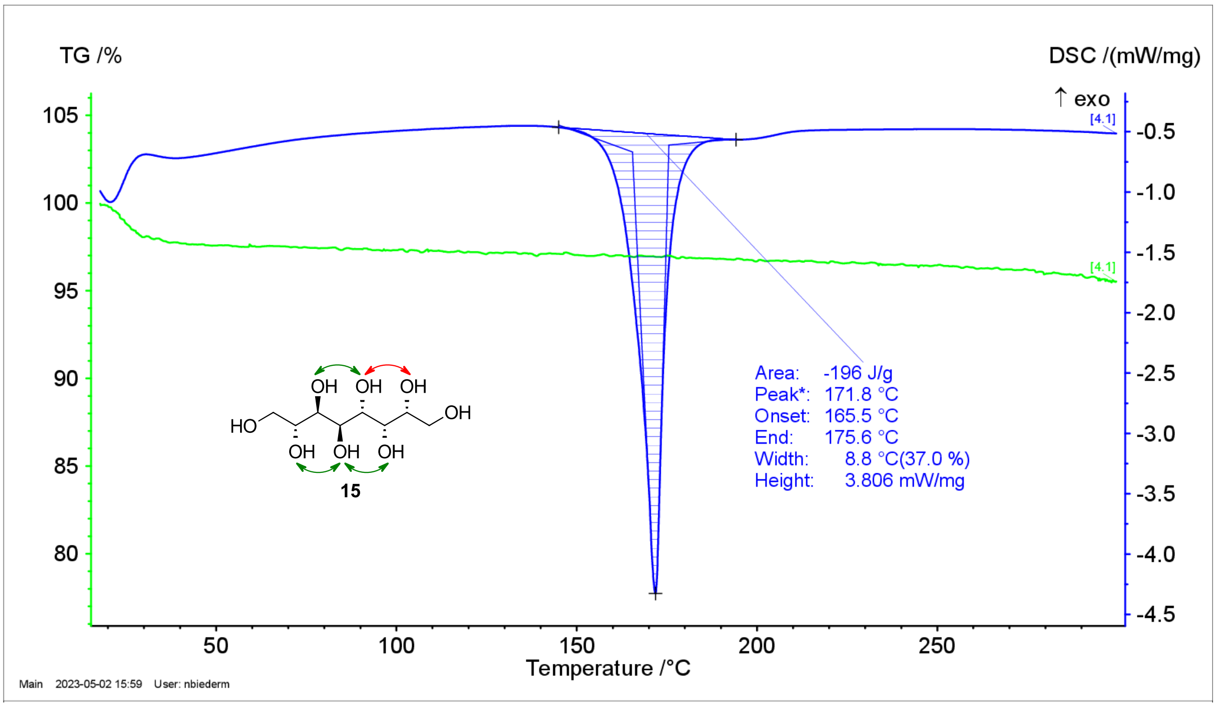

Figure S27. STA measurement of D-*erythro*-L-*gluco*-octitol (**15**)

References

1. Stanetty C ,Baxendale IR (2015) Eur J Org Chem 2015:2718
